# Cohort profile: BioCaPPE (Biomarkers of Prostate Cancer/Prevention and Environment) – a Canadian multicentre prospective study of lifestyle and candidate biomarkers in relation to prostate cancer risk

**DOI:** 10.1136/bmjopen-2025-111118

**Published:** 2026-05-28

**Authors:** Hanane Moussa, Roxane Tourigny, Karine Robitaille, Vanessa Bussières, Jonathan Fadel, Farah Ben Souilah, Lamoussa Diabaté, Hélène Hovington, Laurence Bettan, Louis Lacombe, Thierry Dujardin, Michele Lodde, Paul Toren, Armen Aprikian, Fred Saad, Michel Carmel, Claudio Jeldres, Benoit Lamarche, Alain Bergeron, Yves Fradet, Vincent Fradet, Network BIOCaPPE, Armen Aprikian

**Affiliations:** 1 Faculty of Medicine, Université Laval, Québec City, Quebec, Canada; 2 Oncology Program, CHU de Québec-Université Laval Research Center, Québec City, Quebec, Canada; 3 Centre NUTRISS - Nutrition santé et société, Université Laval, Québec City, Quebec, Canada; 4 Centre de Recherche sur le Cancer, Universite Laval, Quebec, Quebec, Canada; 5 Institut sur la Nutrition et les Aliments Fonctionnels, Université Laval, Québec City, Quebec, Canada; 6 Centre Intégré de Cancérologie, CHU de Québec-Université Laval, Québec City, Quebec, Canada; 7 McGill University Health Centre, Montreal, Quebec, Canada; 8 Université de Montréal, Centre Hospitalier de l’Université de Montréal, Montreal, Quebec, Canada; 9 Centre de Recherche du CHUS, Université de Sherbrooke, Sherbrooke, Quebec, Canada

**Keywords:** Risk Factors, Quality of Life, Primary Prevention, Prostate disease

## Abstract

**Abstract:**

**Purpose:**

The BioCaPPE (Biomarkers of Prostate Cancer/Prevention and Environment) study is a multicentre prospective observational cohort designed to identify biomarkers associated with prostate cancer (PCa) risk that may be modifiable through lifestyle factors. This paper describes the cohort, along with the data and bio-samples available for future studies in PCa risk assessment.

**Participants:**

Canadian men at risk of PCa were enrolled based on one of two criteria (1) negative first prostate biopsy within 6 months from enrolment (Group 1); or (2) a prostate-specific antigen (PSA) blood level between 2.5 and 10 ng/mL without prior prostate biopsy (Group 2). At baseline, blood samples and comprehensive data were collected. PCa incidence and lifestyle factors were updated for all participants over 2 years, with extended follow-up for those who provided additional consent.

**Findings to date:**

Recruitment was conducted across four health centres in Quebec, Canada. A total of 2053 men were enrolled—1499 in Group 1 and 554 in Group 2. All participants completed the initial visit, which included collection of medical and family history, anthropometric measurements, demographic information, dietary and alcohol intake, physical activity, tobacco use, medication use, and quality of life assessments, and candidate biomarker measurements. At the 2-year mark, 7.2% of participants had developed PCa; this figure has since increased to 15.3% (median follow-up: 6.1 years). Additionally, 84% (n=1718) consented to ongoing annual follow-up.

**Future plans:**

This large, prospective cohort of men at risk of PCa offers valuable resources for risk stratification and primary prevention. The BioCaPPE biosamples and data are available to support the identification of lifestyle-related biomarkers associated with PCa risk in this population.

**Trial registration number:**

ClinicalTrials.gov Identifier: NCT03383016.

**STRENGTHS AND LIMITATION OF THIS STUDY**

- As with other prevention studies, the Biomarkers of Prostate Cancer/Prevention and Environment (BioCaPPE) cohort may have limited external validity, as participants tended to have higher socioeconomic status and were less likely to smoke compared with the general population.
- The cohort may be subject to social desirability bias due to reliance on self-reported data for lifestyle and quality-of-life measures.
- Prostate cancer (PCa) incidence has been rigorously documented through annual reviews of participants’ medical records, conducted with their consent.
- A robust sample of 2053 men at high risk of PCa was recruited from four centres across the province of Quebec.
- Validated instruments were used to assess biomarkers, diet, physical activity and quality of life. Anthropometric measurements were standardised across study sites by trained research nurses.

## Introduction

 Prostate cancer (PCa) is the most common non-skin cancer in Canadian men.^1^ Lifestyle factors, primarily diet and physical activity, have been associated with PCa risk in the general population.^2 3^ These environmental exposures are modifiable, offering promising avenues for targeted prevention strategies. Given PCa’s long latency period^4^ and the multiple factors affecting its diagnosis, including referral and prognostic selection biases, investigating risk factors with high-risk populations is essential. However, evidence linking lifestyle exposures to PCa risk in such populations remains limited. Case-control studies have reported associations between dietary habits and PCa risk,^5 6^ while one prospective study identified a link with lethal PCa risk.^7^ Although these findings suggest a potential relationship, the evidence remains inconsistent. Physical activity has been broadly associated with improved general health. A systematic review by the European Association of Urology Section of Oncological Urology concluded that physical activity may be beneficial for the prevention and recurrence of PCa.^8^ In contrast, studies indicating a harmful association are rare.^9^

Given the lack of consistent evidence, no definitive recommendations can currently be made regarding lifestyle and PCa risk. In response to this gap, our team established a prospective cohort of men at risk of PCa, with the objective of identifying lifestyle factors and associated biomarkers that could support risk stratification. This approach aims to inform strategies for the prevention, or at minimum the delay, of PCa development.

The primary objective of this ongoing observational study is to evaluate the association between lifestyle-related candidate biomarkers and the risk of PCa. Secondary objectives include: establishing direct relationships between environmental exposures—such as diet and physical activity—and PCa risk; examining the associations between lifestyle habits and the candidate biomarkers; exploring the links between the candidate biomarkers and inflammation in normal prostate tissue; and assessing the relationship between direct measures of environmental exposures and quality of life of men at high risk of PCa.

Extensive data and biological samples were collected to enable precise characterisation of study participants and to establish a comprehensive biobank for future research on PCa risk assessment. The baseline biological samples, along with prospectively collected data, are available to identify biomarkers related to modifiable lifestyle factors, PCa risk and quality of life in men at risk of PCa. Here, we describe this prospective Canadian cohort, which represents a unique opportunity to develop tools for risk stratification and primary prevention of PCa.

## Cohort description

### Design and setting

The BioCaPPE (Biomarkers of Prostate Cancer/Prevention and Environment) study is a multicentre prospective cohort study conducted across four university-affiliated health centres in the province of Quebec: Centre Hospitalier Universitaire de Québec-Université Laval (CHUQc-UL), Centre Hospitalier de l’Université de Montréal (CHUM), Centre Hospitalier Universitaire de Sherbrooke (CHUS) and McGill University Health Center (MUHC).

Recruitment efforts included poster campaigns and regular outreach to urologists at participating hospital clinics. Additionally, many family physicians were informed about the study and referred eligible participants. The first participant was enrolled on 1 August 2013, and recruitment was completed on 22 September 2020, with a total of 2053 men enrolled.

## Study population

Eligible participants for this prospective cohort study included two groups of men considered at risk for PCa. Group 1 comprised men who had undergone a first negative prostate biopsy within 6 months prior to enrolment. Group 2 included men with prostate-specific antigen (PSA) levels between 2.5 and 10 ng/mL and no prior prostate biopsy. Participants were required to be aged 18 years and older. Exclusion criteria included a history of prostate biopsy (except within 6 months of enrolment for Group 1), any personal history of PCa, the presence of adenocarcinoma identified during transurethral prostatic resection (clinical stages T1a and T1b), inability to provide informed consent, and failure to complete the baseline assessment.

### Ethics approval and consent to participate

The study protocol and associated biobank were approved by the Institutional Review Boards of CHUQc-UL (2012-1110, 2012-1111), CHUM (13.236), CHUS (2014-704) and MUHC (2015-1149). Informed consent was obtained from all participants for both study participation and biobanking of biological specimens and data for future research. The initial consent period was limited to 2 years, consistent with the study design, which was statistically powered for a 2-year follow-up. Participants who agreed to extended follow-up also signed an additional consent for a lifelong annual follow-up, conducted centrally at CHUQc-UL, the coordinating centre for the study.

## Data collection, Managing and Monitoring

Data collection as conducted at research centres by trained research nurses, research professionals and medical trainees. Except for web-based instruments such as the web-based food frequency questionnaire (webFFQ), all original paper questionnaires were scanned, validated by a research professional and securely stored in locked cabinets. Data were stored on the CHUQc-UL network according to Standard Operating Procedures (SOPs) to ensure confidentiality. WebFFQ data were stored on secure servers at Université Laval. Access to the data is restricted to the principal investigator and designated research staff directly involved into the study. The research team has conducted regular monitoring of data collection follow-up procedures.

### Study baseline

During the initial visit, participants completed validated questionnaires covering dietary habits, physical activity, quality of life, sociodemographic characteristics and clinical history. Additional information was collected on medication use, tobacco and alcohol consumption, lifetime sexual history, and personal and family medical history, with a particular focus on cancer. The questionnaires used are detailed in the Data Collection section below. Clinical details were obtained through participant self-report and medical chart review, corroborated by pharmacy records. These data were carefully reviewed by research staff to minimise missing information. Blood samples were collected from all participants, and urine samples were obtained from a sub-cohort for biomarker analysis. Historical PSA testing data were also collected. Baseline PSA was defined as the last PSA test result before enrolment for all patients with the specification that it must be before prostate biopsy session for Group 1 participants. Anthropometric measurements were performed by trained research nurses and professionals using standardised protocols.

### Clinical Follow-up

One year after study enrolment, all participants were contacted via telephone or e-mail to gather information on new medical events, including new PCa diagnosis and additional PCa-related assessments (such as PSA testing, prostate biopsies or imaging procedures). In parallel, participant’s medical records were reviewed to identify new clinical developments and confirm any PCa diagnoses. Update on medications taken since study entry was obtained through participant self-reports and verified using pharmacy records.

At the 2-year follow-up, all participants were contacted via telephone or email to update information on medical events. Changes in medication and lifestyle, particularly diet and physical activity, were recorded. The initial protocol for Group 1 included a recommendation for a prostate biopsy 2 years after enrolment, based on the Reduction by Dutasteride of Prostate Cancer Events (REDUCE) trial,^10^ a large-scale trial available at that the time of BioCaPPE study design. However, shortly after the study’s launch, the practice of routine re-biopsy at 2 years for men with a previously negative result was progressively discontinued by clinicians. Instead, an individualised assessment was conducted for all participants based on clinical guidelines^11^,^14^ and considering lower urinary tract symptoms, PSA kinetics, digital rectal examination of the prostate, familial cancer history, age, estimated remaining lifespan. The decision to order a prostate biopsy was a shared decision with the participant at the discretion of the treating urologist based on an elevated suspicion of prostate cancer on follow-up.

Prostate imaging with ultrasound only without biopsy was allowed but was not performed by clinicians in this study. Prostate imaging with MRI, which progressively became available during the recruitment of this study, was ordered as a shared decision with the patient at the discretion of the treating urologist based on clinical preferences and absence of MRI contraindications such as non-MRI-compatible implanted devices, ferromagnetic foreign bodies, gadolinium contrast allergy or claustrophobia. MRI data were collected when available.

Regardless of diagnosis status, medical history follow-up continued until the scheduled end of the study, and beyond for those who consented to long-term follow-up.

During the early years of the study, evolving controversies surrounding PSA testing and the decline of systematic ordering of prostate biopsy sessions among men with elevated PSA created an opportunity to recruit a second group, i.e. men with a serum PSA level between 2.5 and 10 ng/mL and who opted against immediate biopsy (Group 2). Recruitment of this group began on 11 November 2015 and concluded on 22 September 2020.

For participants who consented to additional long-term follow-up, data on new medical events, medication use and lifestyle changes were collected annually until death or withdrawal of consent. The same validated questionnaires administered at baseline were used throughout for long-term follow-up period. Long-term data collection is still ongoing.

### PCa incidence

PCa incidence was determined mainly through systematic transrectal ultrasound-guided 10–12 core prostate biopsy session, which was the guideline-recommended method for pathological diagnosis and grading of PCa at the time of recruitment.^12 15 16^ Although prostate MRI was not yet integrated into the standard diagnostic pathway in Canada during the majority of the recruitment period, prostate MRI evaluations were allowed in this study. Some patients thus also had targeted biopsy (using cognitive fusion or ultrasound-MRI fusion technology) in addition to systematic biopsy sampling. Data about ultrasound technique or biopsy fusion approach were not collected as this study was designed before practice changing trials about MRI.^17^ Tumour grading was initially based on the Gleason score and, transitioned in 2016, to the International Society of Urological Pathology (ISUP) classification.^18^ When available in medical records, TNM (tumor, node, metastasis) staging was also documented using the eighth edition of the American Joint Committee on Cancer (AJCC) classification.^19^

### Diet

Dietary intake over the preceding month was assessed at baseline using a semi-quantitative webFFQ, specifically developed and validated for Quebec residents.^20 21^ The webFFQ includes extensive photographic representations of portion sizes using standardised dishes, enhancing its precision and approaching the level of quantitative dietary assessment. It comprises 136 questions and 40 sub-questions across eight food groups, aligned with the categories of the 2019 edition of Canada’s Food Guide. In other cohorts, the median completion time of the webFFQ has been 42 min. The questionnaire also captures alcohol consumption, dietary supplement use and provides data on 446 food items and 119 nutrients. Additionally, information on meal substitute consumption (eg, liquids and bars) was collected using a separate questionnaire developed by the research team (see online supplementary material).

### Physical activity

Leisure time physical activity over the preceding year was assessed using the brief Godin Leisure-Time Exercise Questionnaire,^22 23^ that was subsequently modified^24^ and validated.^25^ Its concise format facilitates participant completion while still enabling the calculation of key exercise variables, including duration across different intensity categories.

### Quality of life

General quality of life was assessed using two validated questionnaires: the Hospital Anxiety and Depression Scale (HADS)^26^ and the 36-Item Short Form Survey (SF-36).^27^ The HADS is a 14-item questionnaire designed to evaluate the severity of anxiety and depression symptoms, while the SF-36 is a widely used tool for assessing health-related quality of life. French-language versions of both questionnaires have been previously validated.^28 29^

PCa-related quality of life, including urinary symptoms, associated quality of life and erectile dysfunction, was evaluated using two validated questionnaires: the International Prostate Symptom Score (IPSS)^30 31^ and the Sexual Health Inventory for Men (SHIM).^32^ The IPSS includes seven items assessing urinary symptoms severity and one item evaluating the impact on quality of life. The SHIM consists of five items measuring the severity of erectile dysfunction symptoms.

### Other variables

Additional variables were collected using questionnaires adapted from the PROCURE Research Network^33^ (see online supplementary material). These included house location history, volunteer activities in the past year, employment status and history, ethnicity, personal medical history, family history of cancer, exposure to secondhand smoke, smoking history, alcohol consumption during youth, physical activity during youth, weight change history and symptoms of andropause. Male pattern baldness and sleep disturbances were assessed using validated questionnaires.^34 35^ Experience and decisional regret related to PSA testing and prostate biopsy were captured using a questionnaire adapted from PiCTure Study^36^ (see online supplementary material). Information on circumcision, vasectomy, sexual behaviours, sexual relationships and history of sexually transmitted infections (both personal and partner-related) was collected using a questionnaire adapted from the work of Dr E. Franco’s team^37 38^ (see online supplementary material). Anthropometric data—including waist and hip circumference, height, weight and five-point skinfold measurements (triceps, biceps, subscapular, suprailiac and calf)—were collected by trained research nurses or professionals using standardised procedures.

### Biosamples collection

Blood biosamples were collected by venipuncture at baseline from all participants, processed according to stringent SOPs, aliquoted and stored at –80°C. Although fasting was encouraged, it was not mandatory. The time of venipuncture, usually early morning, was recorded. Aliquots included whole blood, plasma, serum, buffy coat and red blood cells.

First-stream urine sample was also collected at baseline, centrifuged to remove cells and debris, and the clarified urine was aliquoted and immediately stored at –80°C. All procedures adhered to rigorous SOPs to ensure sample integrity.

### Statistical analysis

In the descriptive analysis, categorical variables are presented as counts and percentages, while continuous variables are reported as mean±SD (standard deviation) and median with interquartile range (IQR). Comparisons between the two groups were conducted using the χ² test for categorical variables and the Wilcoxon test for continuous variables. All statistical analyses were performed using Statistical Analysis System (SAS) software, Version 9.4. (Copyright SAS Institute Inc). Two-sided tests were used, with a p value <0.05 considered as statistically significant.

As expected, the incidence of PCa diagnosis is higher in group 2. Thus, analyses of the whole BioCaPPE cohort will be stratified by group. If a similar risk ratio is observed for the exposure variable (eg, baseline biomarker) with the prostate cancer incidence outcome, then both groups will be combined. If not, stratified analyses will be presented. Also, except for cases when the variable is a mediator in the expected causal pathway based on directed acyclic graphs, all analyses will adjust for age and baseline PSA level as these are established prostate cancer risk factors.

### Patient and public involvement

Patients and members of the public were not involved in the design, conduct, reporting or dissemination plans of this study, as it was initiated prior to the widespread adoption of patient partnership practices in research. However, the research team acknowledged the importance of patient and public involvement and is committed to integrating patient partners in future studies stemming the BioCaPPE cohort, particularly in the context of prostate cancer prevention.

## Findings to date

**Figure 1:**
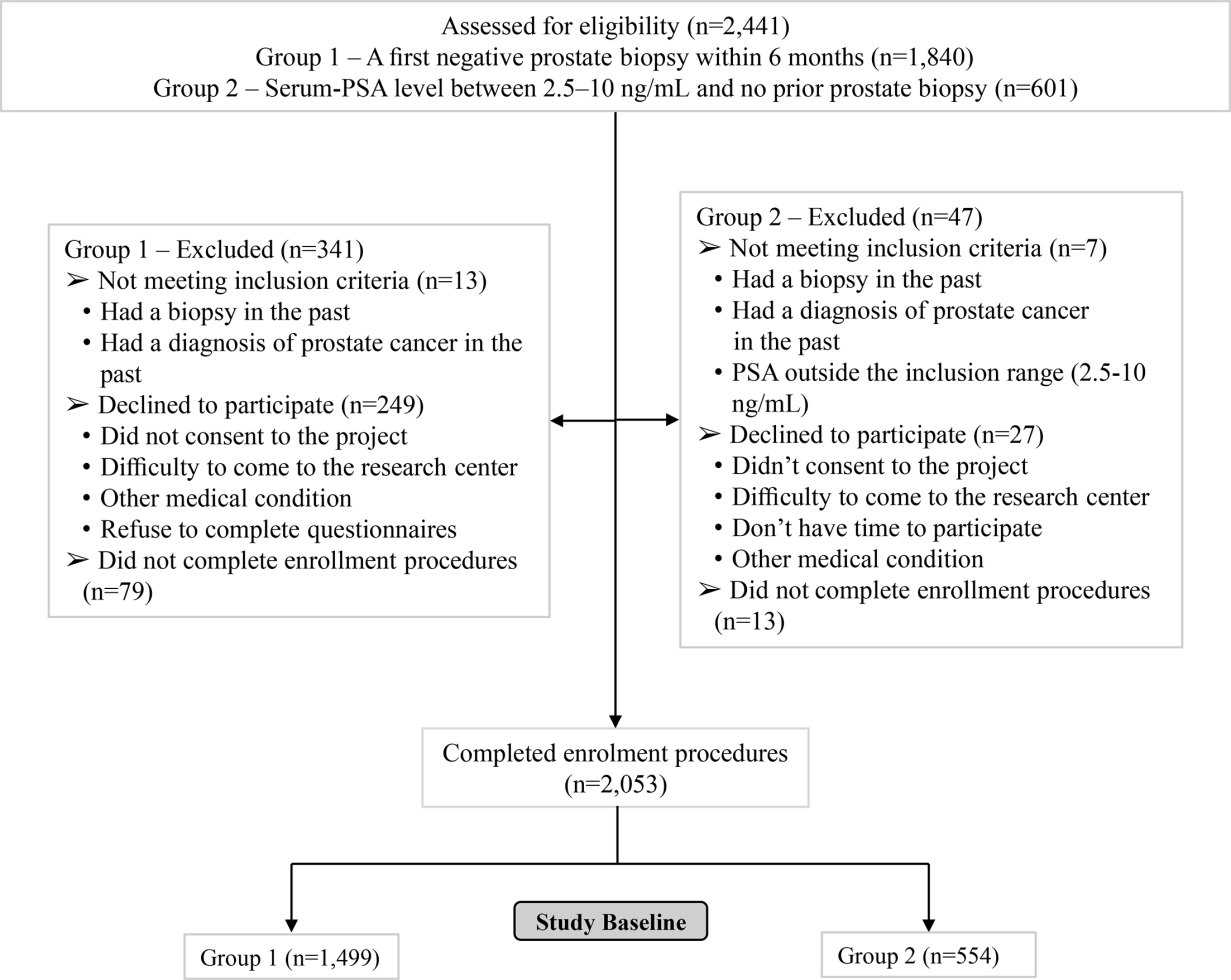
Study flowchart. Legend: For Group 1, a negative biopsy result was defined as the absence of cancer; however, findings such as atypical small acinar proliferation (ASAP) and prostatic intraepithelial neoplasia (PIN) were considered acceptable. For both Group 1 and Group 2, prior transurethral resection of the prostate (TURP) was accepted only if the pathological diagnosis was negative. All participants provided written informed consent and signed a contact permission form, along with an authorisation for disclosure, to confirm their eligibility for the study. PSA, prostate-specific antigen.

The research team assessed the eligibility of 2441 men—1840 in Group 1 and 601 in Group 2 (figure 1). Of these, 2053 (1499 in Group 1 and 554 in Group 2) were enrolled and completed the initial visit. In addition, five candidate biomarkers were measured: serum insulin-like growth factor 1 (IGF-1), oxidised low-density lipoprotein (oxLDL), adiponectin, fatty acid profiles from erythrocyte membranes and selected single nucleotide polymorphisms (SNPs) in genes involved in steroidogenesis. Participation proportions at baseline were 81% for Group 1 and 92% for Group 2.

Baseline characteristics of participants are presented in table 1. Most participants were white men of European ancestry (78.7%). The mean PSA level across all participants was 5.5 ng/mL. A family history of PCa was reported by 17.5% of participants for first-degree relatives, 7.0% for second-degree relatives and 4.4% for both. Benign prostatic hyperplasia (52.3%), hypercholesterolaemia (36.1%) and hypertension (34.4%) were highly prevalent, while diabetes (10.0%), myocardial infarction (5.5%), cardiac insufficiency (3.3%) and arteriosclerosis (2.2%) were less common.

**Table 1:** Baseline characteristics of study participants, stratified by risk group

Baseline characteristics	All participants (n=2053)	Group 1* (n=1499)	Group 2* (n=554)	P value†
**PSA (ng/mL**)				**<0.001**
Mean (SD)	5.5 (3.1)	5.7 (3.4)	4.9 (2.0)	
Median (Q1–Q3)	5.0 (3.8–6.6)	5.2 (4.0–6.9)	4.5 (3.4–5.9)	
Missing data (n, %)	3 (0.1)	1 (0.1)	2 (0.4)	
**Family history of prostate cancer, n (%**)				0.061
First degree relatives only	359 (17.5)	243 (16.2)	116 (20.9)	
Second degree relatives only	144 (7.0)	102 (6.8)	102 (7.6)	
First and second degree relatives	90 (4.4)	65 (4.3)	25 (4.5)	
None	1414 (68.9)	1055 (70.4)	359 (64.8)	
Missing data	46 (2.2)	34 (2.3)	12 (2.2)	
**Comorbidities, n (%**)				
Benign prostatic hyperplasia	1073 (52.3)	834 (55.6)	239 (43.0)	**<0.001**
Cholesterol	742 (36.1)	551 (36.8)	191 (34.5)	0.329
Hypertension	706 (34.4)	502 (33.5)	204 (36.8)	0.160
Diabetes	206 (10.0)	147 (9.8)	59 (10.7)	0.577
Myocardial infarction	112 (5.5)	72 (4.8)	40 (7.2)	**0.033**
Cardiac insufficiency	67 (3.3)	38 (2.5)	29 (5.2)	**0.002**
Arteriosclerosis	45 (2.2)	29 (1.9)	16 (2.9)	0.191
Missing data	46 (2.2)	34 (2.3)	12 (2.2)	
**BMI (kg/m2†**)				0.856
Mean (SD)	28.2 (4.5)	28.2 (4.5)	28.2 (4.5)	
Median (Q1–Q3)	27.6 (25.2–30.4)	27.6 (25.2–30.4)	27.6 (25.1–30.5)	
Missing data (n, %)	12 (0.6)	9 (0.6)	3 (0.5)	
**Waist circumference (cm**)				0.898
Mean (SD)	100.6 (11.7)	100.6 (11.5)	100.6 (12.1)	
Median (Q1–Q3)	99.5 (93.0–107.0)	99.5 (93.0–107.0)	100.0 (92.5–108.0)	
Missing data (n, %)	21 (1.0)	10 (1.0)	11 (2.0)	
**Age (years**)				**<0.001**
Mean (SD)	63.2 (7.5)	62.8 (7.2)	64.4 (8.2)	
Median (Q1–Q3)	64.0 (58.0–68.0)	63.0 (58.0–68.0)	65.0 (59.0–70.0)	
Missing data (n, %)	0 (0)	0 (0)	0 (0)	
**Marital status, n (%**)				0.725
Married or common-law	1540 (75.0)	1121 (74.8)	419 (75.6)	
Single	470 (22.9)	346 (23.1)	124 (22.4)	
Missing data	43 (2.1)	32 (2.1)	11 (2.0)	
**Education level, n (%**)				0.714
Secondary school or less	515 (25.1)	383 (25.6)	132 (23.8)	
Postsecondary diploma	607 (29.6)	441 (29.4)	166 (30.0)	
University degree	885 (43.1)	641 (42.8)	244 (44.0)	
Missing data	46 (2.2)	34 (2.3)	12 (2.2)	
**Annual income, n (%**)				**0.030**
<$60 000	846 (41.2)	641 (42.8)	205 (37.0)	
$60 000−$100 000	554 (27.0)	385 (25.7)	169 (30.5)	
>$100 000	522 (25.4)	383 (25.6)	139 (25.1)	
Prefer not to answer	85 (4.1)	56 (3.7)	29 (5.2)	
Missing data	46 (2.2)	34 (2.3)	12 (2.2)	
**Ethnicity, n (%**)				**<0.001**
European ancestry white	1615 (78.7)	1203 (80.3)	412 (74.4)	
Non-European white‡	144 (7.0)	86 (5.7)	58 (10.5)	
Indigenous peoples of Canada	3 (0.2)	1 (0.1)	2 (0.4)	
African (black)	13 (0.6)	9 (0.6)	4 (0.7)	
Asian	6 (0.3)	3 (0.2)	3 (0.5)	
Pacific Islander	1 (0.1)	1 (0.1)	0 (0)	
Mixed	80 (3.9)	66 (4.4)	14 (2.5)	
Unknown	142 (6.9)	95 (6.3)	47 (8.5)	
Missing data	49 (2.4)	35 (2.3)	14 (2.5)	
**Smoking status, n (%**)				0.238
Current smoker	171 (8.3)	134 (8.9)	37 (6.7)	
Former smoker	1037 (50.5)	755 (50.4)	282 (50.9)	
Never	798 (38.9)	575 (38.4)	223 (40.3)	
Missing data	47 (2.3)	35 (2.3)	12 (2.2)	
**Alcohol use§ (g/day**)				0.098
Mean (SD)	14.7 (17.7)	14.3 (16.9)	15.8 (19.6)	
Median (Q1–Q3)	9.4 (2.0–18.9)	8.8 (1.9–18.4)	10.6 (2.8–20.5)	
Missing data (n, %)	230 (11.2)	167 (11.1)	63 (11.4)	
**Physical activity¶, n (%**)				0.837
Inactive (0 min/week)	483 (23.5)	359 (24.0)	124 (22.4)	
Not sufficiently active (1–149 min/week)	760 (37.0)	552 (36.8)	208 (37.6)	
Active (150–300 min/week)	349 (17.0)	256 (17.1)	93 (16.8)	
Very active (≥300 min/week)	410 (20.0)	294 (19.6)	116 (20.9)	
Missing data	51 (2.5)	38 (2.5)	13 (2.4)	

* Group 1: First negative biopsy ≤6 months prior to enrolment; Group 2: PSA level between 2.5 and 10 ng/mL with no prior biopsy.

† P values were calculated using the χ² test for categorical variables and the Wilcoxon test for continuous variables, comparing Group 1 and Group 2. Bold values indicate statistically significant (p<0.05).

‡ Origin from North Africa (eg, Algeria, Tunisia), the Middle East (eg, Egypt, Iran) or South Asia (eg, Afghanistan, India).

§ At the time of the study, Canadian guidelines for men aged 18 years and older recommended a maximum alcohol intake of 40 g per day (equivalent to three drinks per day, with a maximum of 15 drinks per week).

¶ Health Canada and WHO guidelines for adults aged 18 years and older recommend engaging in 150 and 300 min of moderate to vigorous physical activity per week.

BMI, Body Mass Index; PSA, prostate-specific antigen.

Given the significant difference between groups at baseline in some cardiovascular comorbidities, we tested the hypothesis that these comorbidities may be used by clinicians to decide against performing a prostate biopsy. None of the baseline comorbidities were individually associated with time to a prostate biopsy session (all p>0.5). We also tested a common definition of major cardiovascular events (MACE) in cardiovascular trials, where either myocardial infarction or stroke is considered exposed. MACE was also not associated with risk of prostate biopsy (HR 1.12, 95% CI 0.86 to 1.15, p=0.42). It is thus unlikely that cardiovascular comorbidities were a significant factor influencing the biopsy decision.

The mean body mass index (BMI) was 28.2 kg/m^2^, and the mean waist circumference was 100.6 cm. The average age of participants at enrollment was 63.2 years. Most participants (75.0%) were married or in a common-law union. Nearly half of participants (43.1%) held a university degree, compared with 32.9% in the general Canadian population,^39^ and the majority (52.4%) reported an annual household income of $60 000 or more.

Income differed across study groups (χ² p=0.03). Among patients recruited at CHUQc-UL (Quebec City), 62% were in Group 1 and 38% in Group 2. At CHUS (Sherbrooke), 94% were in Group 1 and 6% in Group 2. When both Montreal sites are aggregated (CHUM and CUSM), 81% were in Group 1 and 19% in Group 2. Regional differences in annual income are observed in the general population. Similar to the χ² test presented in table 1, in a crude multinomial model, the overall effect of group on annual income was statistically significant (p=0.035). After adjusting for city, the overall effect of group was no longer significant (p=0.293), and the ORs for Group 2 versus Group 1 shifted toward the null. Specifically, the OR for income category 2 ($60 000–$100 000) vs 1 (<$60 000) decreased from 1.37 to 1.22 (−11.2%), and the OR for income category 3 (>$100 000) vs 1 decreased from 1.14 to 1.05 (−7.5%). These changes indicate that city acts as a confounder of the association between group and income, particularly for the comparison between income categories 2 and 1.

Only 8.3% of participants were current smokers, and 38.9% had never smoked—lower than the national rate of current smokers (12.0%).^40^ Average alcohol consumption was 14.7 g/day, equivalent to approximately one drink per day. Among participants who consumed alcohol (99% of participants), the average alcohol intake was 14.8 g/day, below the national recommendation at the time of study enrolment (three drinks per day or 15 per week for men).^41^

More than a third (37.0%) of participants met the national guideline of at least 150 min of moderate to vigorous physical activity per week,^42^ which is lower than the proportion observed in the general Canadian adult population (49.2%).^43^

Significant difference between Group 1 (participants with a prior negative prostate biopsy) and Group 2 (participants without prior biopsy) were observed for PSA level (p<0.001), prevalence of benign prostatic hyperplasia (p<0.001), myocardial infarction (p=0.033), cardiac insufficiency (p=0.002), age (p<0.001), annual income (p=0.030) and ethnicity (p<0.001).

The number of participants recruited at each site is detailed in table 2, with the majority enrolled at CHUQc-UL. The 2-year data collection phase concluded in September 2022, while the long-term follow-up has continued thereafter. Of the 2053 participants who initially consented to participate, 1957 (95%) remained enrolled at the 2-year follow-up, and 1718 (84%) agreed to continue with the long-term follow-up. Table 3 outlines the specific data collected at each timepoint throughout the study.

**Table 2:** Recruitment and long-term follow-up of participants by site

Centres	Baseline, n (%)		2-year, n (%)		≥3 years, n (%)	
	Group 1* (n=1499)	Group 2* (n=554)	Group 1* (n=1421)	Group 2* (n=536)	Group 1* (n=1230)	Group 2* (n=488)
CHUQc-UL	705 (47.0)	441 (79.6)	692 (48.7)	434 (81.0)	667 (54.2)	425 (87.1)
CHUS	416 (27.8)	25 (4.5)	406 (28.6)	25 (4.7)	372 (30.2)	22 (4.5)
CHUM	303 (20.2)	33 (6.0)	259 (18.2)	32 (6.0)	175 (14.2)	26 (5.3)
MUHC	75 (5.0)	55 (9.9)	64 (4.5)	45 (8.4)	16 (1.3)	15 (3.1)

* Group 1: First negative biopsy ≤6 months prior to enrolment; Group 2: PSA level between 2.5 and 10 ng/mL with no prior biopsy.

CHUM, Centre Hospitalier Universitaire de Montréal; CHUQc-UL, Centre Hospitalier Universitaire de Québec-Université Laval; CHUS, Centre Hospitalier Universitaire de Sherbrooke; MUHC, McGill University Health Center.

**Table 3:** Data collection at each timepoint

		Baseline	1-year	2-year	≥3-year
**Clinical variables**					
Anthropometry	Height, weight, waist and hip circumferences, 5-point skinfold thickness (triceps, biceps, iliac, scapular and calf).	X			
PSA	Total and free PSA, and density.	X	X	X	X
Prostate cancer	Grade, stage, NCCN risk, and prostate volume.	At prostate cancer diagnosis			
Medication	Complete medication profile from pharmacy records.	X	X	X	X
**Self-reported variables**					
Sociodemographic	Age, marital status, education, income, employment status and occupation, habitation history, volunteer work, ethnicity.	X			
Medicals	History of prostate and other cancers, prostatic and other diseases, hair loss, sleep problems, weight changes history, andropause-related symptoms, circumcision/vasectomy, sexually transmitted diseases, and experience with PSA/biopsy.	X		X	X
Lifestyle factors	Physical activity (leisure-time and during youth), smoking status and secondhand smoke exposure, alcohol intake including during youth, and sexual behaviours.	X		X	X
Diet	Food frequency questionnaire, and dietary supplements.	X		X	X
General quality of life	Depression, anxiety, physical and social functioning, physical and emotional role, mental health, vitality, global pain and general health.	X			
Quality of life specific to PCa	Sexual dysfunction, urinary problems and quality of life in relation to urinary symptoms.	X			
**Biological variables**					
Biomarkers	Fatty acid profiles, oxLDL, IGF-1, adiponectin, hormone targeted-SNPs	X			

SNPs: Single Nucleotide Polymorphisms.IGF-1, insulin-like growth factor 1; NCCN, National Comprehensive Cancer Network; oxLDL, oxidised low-density lipoprotein; PCa, prostate cancer; PSA, prostate-specific antigen.

In addition to the extensive dataset collected at various timepoints throughout the study, a range of biospecimens was obtained at baseline for biomarker analysis and future research purposes. These include blood and urine samples, as well as formalin-fixed paraffin-embedded (FFPE) prostate needle biopsy specimens (table 4).

**Table 4:** Biosamples available in the BIOCaPPE biobank

Biosamples	Method of storage	Available quantities
	Whole blood	1.2 mL*
	Plasma	9 mL*
Blood	Serum	8.3 mL*
	Buffy coat	0.4–3.5 mL*
	Red blood cells	1.5 mL*
Urine	Clarified (first stream)	20 mL*
Prostate needle biopsies	FFPE	12 biopsy cores†

* Approximate total volume available in the biobank at the baseline timepoint.

† Non-targeted biopsies were available for Group 1 only.

BIOCaPPE, Biomarkers of Prostate Cancer/Prevention and Environment; FFPE, formalin-fixed paraffin-embedded.

**Figure 2:**
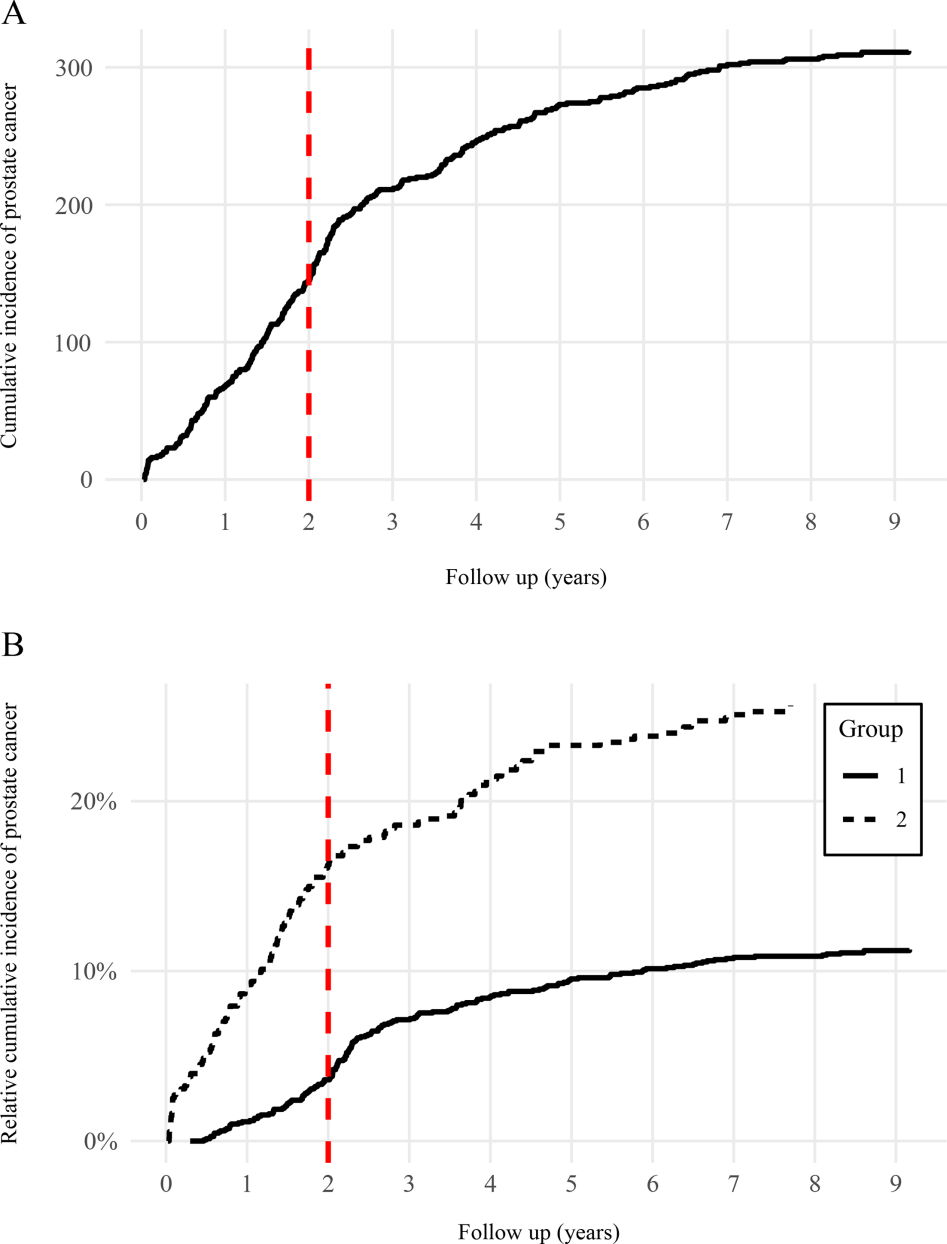
Incidence of prostate cancer cases. Legend: Cumulative incidence of prostate cancer diagnosed in the cohort between 2013 and 2024. (A) Absolute number of cases. (B) Incidence relative to each study group. The Y axis indicates the percentage of participants diagnosed with prostate cancer out of the total number of participants for each group (Group 1 = 1499; Group 2 = 554).

At the most recent follow-up in September 2024, PCa had been diagnosed in 314 participants across both groups. MRI-guided biopsies were used in 23% of PCa diagnoses, with no difference between Groups 1 and 2 nor in ISUP distribution. The cumulative incidence plateaued at approximately 15% after 6 years of follow-up (13.9%) (figure 2A). As expected, the incidence differed between Group 1 and Group 2. During the first 2 years of the study, 7.2% of men developed PCa—3.7% in Group 1 and 16.1% in Group 2. At the 2024 medical information review, the overall incidence had reached 15.3% at a median follow-up of 6.1 years (Q1=4.4, Q3=7.8). In Group 1, the incidence was 11.3% (median follow-up=6.5 years; Q1=4.7, Q3=8.3), while in Group 2, it was 26.0% (median follow-up=5.1 years; Q1=3.2, Q3=6.5) (figure 2B). The distribution of pathological stages differed significantly between the groups (p<0.001), whereas clinical stage distributions were similar (p=0.175). In Group 1, PCa cases were most frequently diagnosed as ISUP Grade Group 1 (50.6%), while in Group 2, ISUP Grade Group 2 as the most common (36.8%) (table 5).

**Table 5:** Description of prostate cancer cases diagnosed during follow-up

Prostate cancer characteristics at diagnostic	Group 1* (n, %) (n=170)	Group 2* (n, %) (n=144)	P value†
**ISUP grade group**			**<0.001**
1	86 (50.6)	45 (31.3)	
2	30 (17.7)	53 (36.8)	
3	30 (17.7)	22 (15.3)	
4	12 (7.0)	8 (5.6)	
5	11 (6.5)	16 (11.1)	
Missing data	1 (0.6)	0 (0)	
**Clinical stage**			0.175
T1	50 (29.4)	27 (18.8)	
T2	29 (17.1)	26 (18.1)	
T3	3 (1.8)	5 (3.5)	
Missing data‡	88 (51.8)	86 (59.7)	

* Group 1: First negative biopsy ≤6 months prior to enrolment; Group 2: PSA level between 2.5 and 10 ng/mL with no prior biopsy.

† P values were calculated using the χ² test for ISUP grade group and Fisher’s exact test for clinical stage. Bold values indicate statistically significant (p<0.05).

‡ Clinical stage was extracted from chart review. This information was not consistently documented in the medical records by treating physicians.

ISUP, International Society of Urological Pathology.

## Relevance, Study Impact and Future Directions

The primary objective of the BioCaPPE cohort study is to identify biomarkers of PCa risk that may be modifiable through lifestyle habits. These biomarkers could serve as surrogate endpoints to evaluate and reinforce the impact of preventive interventions aimed at promoting healthier lifestyles among men at risk of PCa. To our knowledge, BioCaPPE represents the largest multicentric prospective cohort of Canadian men at risk of PCa. This study was initiated slightly before practice changes and eventually clinical guidelines recommending a more selective usage of prostate biopsy. This modern approach involves the patient if a shared decision-making, and considers numerous clinical factors listed above such as symptoms, PSA kinetics, physical examination and familial cancer history.

The context of this study makes it particularly actionable as it recruited participants referred to or at charge of urologists because identified at elevated risk of prostate cancer. Given the typically longer latency of most prostate cancer cases, it is possible that the lifestyle factors (and related biomarkers) may have influenced prostate cancer before this study measured them. This is in part why we expanded the follow-up duration without restriction. In the same line, this study also measures some factors thought to affect metabolism during teenage years, a period hypothesised to affect prostate growth and possibly dysregulation.

Nevertheless, prostate cancer is known as a complex multifactorial disease.^44^ Thus, expanding on Bradford Hill criteria evaluating causality,^45^ and based on the multicausality framework,^46 47^ this study aims to identify risk factors (eg, biomarkers) associated with component causes (eg, lifestyle element). Even if these are identified somewhat ‘downstream’ or closer to disease identification, they still can be considered a contributing factor to causing the disease of interest. What is more, the overarching hypothesis of this study is that the referral and recruitment context in urology clinics makes the study findings actionable for prostate cancer prevention such as trials testing targeted risk-reduction strategies and patient education about their lifestyle.

The study design, prospective with a minimum follow-up of 24 months, includes a comprehensive collection of personal, medical, and lifestyle data, enhances the study’s ability to minimise bias and control confounding factors. By investigating a novel set of biomarkers potentially influenced by lifestyle, the study seeks to improve prediction and prevention of clinically significant PCa. This innovative and unprecedented approach involves participants recruited from multiple university hospital centres across Quebec. Given that some recruitment sites (eg, CHUQc-UL and CHUS) offer centralised urology consultations and biopsy procedures—thereby capturing nearly all eligible participants in their respective regions—and that recruitment was supported by outreach to primary care physicians, we believe this cohort is representative of the population of men at risk of PCa in the province of Quebec, Canada.

Participants at risk of PCa were defined as those with either an initial negative biopsy (Group 1) or a PSA level between 2.5 and 10 ng/mL without a prior biopsy (Group 2). A greater number of participants were enrolled in Group 1 (n=1499) compared with Group 2 (n=554), as many, primarily aged 55–69 years old, underwent biopsy following elevated PSA levels according to the prevailing consensus at time of study initiation. Individuals with a first negative biopsy remain at a 17%–25% risk of being diagnosed with PCa in subsequent years when biopsied more liberally,^10 48^ while those with moderately elevated PSA levels (2.5–10 ng/mL) face a higher risk of 25%–30%.^49 50^ Two randomised controlled trials have demonstrated that PCa prevention is feasible in high-risk individuals through the use of 5α-reductase inhibitors,^10 48^ with biopsies systematically proposed at either 2 years^10^ or 7 years^48^ in these studies.

Over the years, numerous studies have identified biomarkers and risk factors associated with PCa incidence. The first widely used and clinically impactful PCa biomarker is serum PSA.^51^ Following the initial commendation by the US Preventative Services Task Force (USPSTF) against the use of PSA for PCa screening, interest has rapidly grown for several alternative biomarker strategies. These include multiparametric MRI (mpMRI),^52^ various molecular kallikreins forms (PSA is human kallikrein-related peptidase 3), the 4Kscore,^53 54^ the Prostate Health Index (PHI) score,^55 56^ and urinary molecular tests such as PCA3,^57 58^ SelectMDx^59^ and MIPS ExoDx Prostate IntelliScore (EPI).^60^ However, these assays are costly and are not currently covered by the Canadian public healthcare system.

Despite important interest in PCa biomarkers, BioCaPPE study is among the first large-scale study to select biomarkers based on their potential link with modifiable lifestyle factors. The biomarkers evaluated in this study are standardised low-cost tests performed in most laboratories. However, to our knowledge, it is the first time they are formally evaluated in relation with PCa risk. Through the identification of novel biomarkers, we aim to lay the conceptual foundations to develop a new methodology for assessing PCa risk. These modifiable biomarkers could also represent a significant conceptual advancement potentially applicable to cancer prevention strategies for the general population.

From a clinical perspective, validating lifestyle factors through multiple questionnaires enables robust longitudinal comparisons between study groups of men at risk of PCa. It is estimated that 30%–40% of cancers could be prevented by modifying lifestyle and environmental risk factors known to influence cancer incidence.^61^,^63^ In the context of PCa, physical activity and diet are particularly relevant modifiable lifestyle factors for prevention.

The primary challenge in studying lifestyle factors and PCa lies in the inconsistency and limited availability of literature, which often relies on suboptimal methodologies and approaches. We believe that BioCaPPE study offers a unique opportunity to optimise bias control and precision by its prospective design, the substantial size of its cohort, and the use of validated assessment tools. These include a food frequency questionnaire specifically adapted to Quebec residents,^20^ and a physical activity questionnaire that have been previously validated.^22 23^

From a practical standpoint, assessing quality of life in relation to modifiable lifestyle factors and PCa incidence introduces a human-centred dimension. The relevance of this dimension has been previously highlighted^64 65^ and is considered essential in decision-making processes during prostate cancer risk assessment and during active surveillance. Clinical decisions are now recommended to use a shared approach between physician and patient, with a quality-of-life consideration playing a central role.^11^,^14^ Thus, using validated questionnaires, we herein propose a comprehensive observational evaluation that will evolve over an extended period of follow-up and provide an opportunity to explore human-centred factors in the context of prostate cancer risk assessment.

An important strength of this study lies its recruitment timing, which coincides with a major shift in clinical practice regarding PCa diagnosis following the USPSTF evaluations of PCa screening in 2012 and 2018.^66^,^69^ In 2012, USPSTF issued a grade D recommendation against PSA-based screening for PCa.^66^ This decision was largely influenced by the findings of the US-based Prostate, Lung, Colorectal, Ovarian (PLCO) cancer trial, which reported no significant difference in PCa-specific mortality between the screened and control groups over a 15-year follow-up.^70^ However, the PLCO trial notably affected by contamination, as a substantial proportion of men in the control group underwent PSA testing either prior to or during the trial—initially estimated at 40%–50%,^71^ but later revised to approximately 90%.^72^

The grade D recommendation led to a marked decline in referrals to urology for PCa risk assessment and a shift in clinical practice toward more selective criteria for prostate biopsy.^73^ The overarching goal is to reduce overdiagnosis of indolent PCa while ensuring timely detection of clinically significant cases eligible for curative treatment. Current diagnostic strategies now incorporate a comprehensive evaluation of family history and genetic predisposition, PSA kinetics and density, and a range of blood biomarkers when available (see above).^12^ Additionally, the adoption of mpMRI for biopsy selection and the use of ultrasound fusion technologies have significantly enhanced the diagnostic yield of clinically significant PCa.^15 74^

PCa screening recommendations have evolved in response to significant controversy,^14 75^ particularly regarding the limitations of the US-based PLCO trial, which was confounded by substantial contamination in the control group.^70 71^ This contamination diminished the apparent benefit of systematic PSA screening. In contrast, European PCa prevention trials with lower contamination rates in their control arms demonstrated a reduction in PCa-specific mortality associated with PSA screening.^76 77^ Reflecting these divergent findings, the USPSTF revised its recommendations in 2018 to advocate for a shared decision-making approach, encouraging discussion between clinicians and patients about the potential benefits and harms of PSA screening. This approach is now widely endorsed in clinical guidelines.^11^,^14^

BioCaPPE represents one of the first larger-scale, multi-institutional prospective cohorts to be recruited within the context of the modern PCa diagnostic era. Consequently, PCa cases identified within BioCaPPE are expected to reflect contemporary diagnostic practices and to represent more clinically significant disease. We believe that our study captures the evolving landscape of PCa diagnosis in light of the USPSTF guideline changes, offering a unique perspective on their impact.

## Strengths and limitations

The BioCaPPE cohort has limitations that may affect generalisability. As observed in other prevention trials such as Prostate Cancer Prevention Trial (PCPT)^78^ and lifestyle studies, BioCaPPE participants exhibited lower smoking rates^79^ and higher socioeconomic status.^80 81^ The observed between-group income differences in BioCaPPE are probably attributable to variations in recruitment proportions among the different cities. They are unlikely to impact healthcare decisions because Canada has a universal healthcare system. However, annual income can impact lifestyle-related decisions, such as eating higher-quality food elements and practising physical activity. As a result, we will adjust all future modelling testing lifestyle-related questions by centre/city and income level. We will also test for interaction, when relevant.

The reliance on self-reported data for diet, physical activity and quality of life introduces the potential for social desirability bias, whereby participants may have under-reported or over-reported behaviours to align with perceived norms. To mitigate this, questionnaires were administered anonymously, and participants were informed that their responses would not be reviewed by research staff, particularly for sensitive PCa-specific quality-of-life items. Additionally, the study does not include central pathology review of biopsy slides, which may introduce variability in histopathological grading but may better reflect real world practice.

Statistical precision may be limiting the capacity of this study to detect associations of small magnitude. We quantified the statistical power for representative hypotheses involving biomarkers assessed in our cohort. Given the lack of published data defining a clinically meaningful incidence rate ratio for PCa associated with the candidate circulating biomarkers, we based our calculations on statistically meaningful HRs used in previous studies. Using the Cox Proportional Hazards Regression procedure in SAS (alpha=5%), the observed number of PCa events (n=314) and a conservative R² value of 0.20 (to account for correlation between adjustment variables), we evaluated HRs ranging from 0.1 to 1.5. Under these assumptions, the minimum detectable HR is 0.82, corresponding to a statistical power of 88.2%. These calculations demonstrate that the cohort achieves sufficient statistical precision to detect realistic, biologically plausible associations for the biomarkers examined. Similar calculations will be repeated for each new analysis to specify the actual power to the updated data.

The BioCaPPE cohort presents several notable strengths. PCa incidence has been rigorously documented on an annual basis through medical record reviews conducted with participant consent. As of September 2024, the combined cohorts reached a plateau in incidence at 6 years of follow-up, with a cumulative incidence of 13.9% (figure 2A). Group 1, comprising men with prior negative biopsy, showed a cumulative incidence of 10.2%, while Group 2, consisting of men with PSA levels between 2.5 and 10 ng/mL and no prior biopsy, reached 24.2% (figure 2B). These figures indicate that both cohorts have attained sufficient maturity to support the evaluation of preventive interventions based on lifestyle-related biomarkers. The particularly high incidence in Group 2 at 3 years (20%) is especially relevant in the context of the shift away from systematic prostate biopsy.

Additional strengths include the recruitment of an adequate sample of men at risk of PCa from four clinical centres, a strong baseline participation rate (84%). PCa risk and prevention studies are particularly susceptible to biases in disease ascertainment.^82^ To minimise such biases, standardised procedures were implanted across study sites. Validated instruments were used to assess biomarkers, diet, physical activity and quality of life. Anthropometric measurements were performed by trained research nurses, and PSA data were obtained from referring physicians. PCa diagnosis was based on prostate biopsy, the gold standard for histopathological confirmation, using a pragmatic approach to grading.

## Collaboration

The authors of this ongoing study are committed to fostering collaboration and actively seek opportunities to broaden the reach and impact of the BioCaPPE cohort. The datasets used and/or analysed are available from the corresponding author on reasonable request. Access to biological specimens is also possible, subject to approval by the biobank committee and alignment with the proposed research objectives in PCa prevention.

## Conclusion

BioCaPPE is a Canadian prospective cohort study on PCa risk that possesses several unique characteristics. Recruitment began shortly after major changes in clinical practice aimed at reducing overdiagnosis and overtreatment, thereby improving the clinical relevance of PCa diagnoses. The cohort provides a rich source of detailed data on health status, lifestyle habits, quality of life and biomarkers related to diet and physical activity. It is anticipated that findings from this study will enhance risk stratification for PCa and help identify modifiable factors and biomarkers that may become actionable targets. Biological samples collected at baseline, along with longitudinal data, offer valuable opportunities to investigate biomarkers associated with lifestyle, PCa risk and quality of life in men at risk. In the long term, BioCaPPE is expected to serve as a valuable resource for researchers, healthcare professionals, urologists and general practitioners, ultimately benefiting patients through improved risk assessment and personalised prevention strategies.

## Supplementary material

10.1136/bmjopen-2025-111118 online supplemental file 1

## Data Availability

Data are available upon reasonable request.
