# Supplementary material for: Cohort profile: BioCaPPE (Biomarkers of Prostate Cancer/Prevention and Environment) – a Canadian multicentre prospective study of lifestyle and candidate biomarkers in relation to prostate cancer risk
Source: BMJ Open. 2026 May 28;16(5):e111118. doi: 10.1136/bmjopen-2025-111118 (PMC13223634; doi:10.1136/bmjopen-2025-111118)
Supplement: online supplemental file 1 [file bmjopen-16-5-s001.pdf]

Supplementary Material

Fradet V. et al. 2026

QUESTIONNAIRE ON LIFESTYLE

Your participation to the different questionnaires is important to better understand prostate cancer prevention. We appreciate your collaboration and are thankful for it.

To the participant: please indicate time at the beginning: __ __ : __ __
H H : M M

Section to be completed by the supervisor:

Questionnaire completed on: 2 0 1 _ - _ _ - _ _

Under the supervision of: Initials _ _ _ _

Initiales pour l'informatisation

OBLIGATOIRE

1- _ _ _ _ _

1800038077

PERSONAL INFORMATIONS

1. What is your **date of birth** Y Y Y Y - M M - D D

2. Where did you spend **most** of your **childhood** (Under 18)?

○ In Quebec ○ Other - specify:

○ Another province or territory of Canada

For Canada indicate the three first positions of the Postal Code

Postal Code

Year of arrival: **and** Year of departure: If you have lived at this adresse discontinuously, please indicate the total number of years spent at this address: years

3. Where did you spend **most** of your **adult life** ?

○ In Quebec ○ Other - specify:

○ Another province or territory of Canada

For Canada indicate the three first positions of the Postal Code

Postal code

Year of arrival: **and** Year of departure: If you have lived at this adresse discontinuously, please indicate the total number of years spent at this address: years

4. What is your **civil status**?
Check **one** answer

Married or couple de facto	Single	Separated or divorced	Widowed	Member of a religious order
○	○	○	○	○

5. Are you living **alone**? Yes ○ No ○ → If not, with whom?

Check **one** answer

○ Spouse
○ Friend(s)
○ Family member(s)
○ Other - specify:

YOUR EDUCATION, OCCUPATION OR PROFESSION

Your profession is important for our study to evaluate work-related risk factors

6. What is the **highest level** of education that you have **completed**?
   Check one answer

- ◯ Primary (1st to 6th)
- ◯ Secondary (7th to 11th)
- ◯ Commercial course
- ◯ Classical course
- ◯ Professional high school degree
- ◯ Collegial (Cégep or 12th & 13th)
- ◯ University- Certificate
- ◯ University- Baccalaureate
- ◯ University- Master
- ◯ University- Doctorate

7. Have you done **volunteer work** over **the last year**? Yes ◯ No ◯

8. What was your **average family income prior taxation last year** (CAD$)?
   Include your retirement income, if applies:
   Check one answer

from 0 to $19 999$	from 20 000 to $39 999$	from 40 000 to $59 999	from 60 000 to $79 999	from 80 000 to $99 999	More than $100 000	Unknown or ou prefer not to answer
◯	◯	◯	◯	◯	◯	◯

9. Over the **last 12 months**, what was your **employment status**?
   If retired with an employment, check full-time worker or part time worker according to your situation.
   Check one answer

- ◯ Full-time worker
- ◯ Part-time worker
- ◯ Unfit for work (disease or injury)
- ◯ Unemployed

Are you retired? Yes ◯ No ◯

If yes, for how long have you been retired: |__|__| years

If yes, are you :
- ◯ retired with an employment
- ◯ retired without an employment

10. What is your **current occupation**, **career** or **profession**?
    If retired without an employment, go to the next question.

____

How long have you had this **occupation**, **career** or **profession**: |__|__| years

8065038077

Following questions are related to the career or profession you occupied for the longest period of time in your life

11. Which career or profession have you occupied for the longest period of time in your life?

12. Indicate the **start** and **end years** of that career of profession you occupied for the longest period of time in your life:

Starting year: | | | | | Is it your current occupation: ○ Yes ○ No ⇒ If not, ending year: | | | | |

If you have occupied that career or profession discontinuously, please indicate the total number of years occupying it:

Total number of years: | | | years

13. Describe your **tasks** and **status** in that occupation:

What was the **name** of the company or organization? ○ Prefer not to answer

In what **city** is or was that company or organization:

14. Is it or was it an **employment**: ○ Full-time ○ Part-time

15. Is it or was it a **seasonal employment**: ○ Yes ○ No

16. What **work shift or schedule** did you occupy **the longest** in that career of profession?
Check **one** answer

○ Day ○ Evening ○ Night ○ Alternating

17. During this employment, **how many hours** were you working in average **per day** or **per week**?

| | | hours/**day** **or** | | | hours/**week**

18. During this employment, are you or were you **most of the time**: Check **one** answer

○ Standing ○ Seated ○ Alternating standing and seated

19. During this employment, are you or were you **most of the time** Check **one** answer

○ Moving (for example construction worker)
○ Unmoving (for example truck driver, administrative agent)
○ Alternating

20. Was it an employment **mostly**: Check **one** answer

○ Indoor ○ Outdoor ○ Alternating indoor and outdoor ○ In a vehicle

YOUR FAMILY ORIGIN

Following questions concern your family history because of the importance of genetics in prostate cancer.

21. Were you adopted ?

Yes	No	Unknown
◯	◯	◯

22. What is the composition of your family (including deceased members)?

Indicate the number of individuals in the appropriate box:
If none write 0 in all boxes.

	Biological	Adopted
Number of brothers:	⎵⎵	⎵⎵
Number of sisters:	⎵⎵	⎵⎵

23. Were members of your **biological family** (with blood ties) **born in the province of Quebec**?

	Yes	No	Unknown	Maternal family	Yes	No	Unknown	Paternal family	Yes	No	Unknown
Father:	◯	◯	◯	Grandfather:	◯	◯	◯	Grandfather:	◯	◯	◯
Mother:	◯	◯	◯	Grandmother:	◯	◯	◯	Grandmother:	◯	◯	◯

24. Describe the racial background of members of your biological family?
Check all that apply.

	Maternal family		Paternal family	
	Grandfather	Grandmother	Grandfather	Grandmother
European caucasian (white):	◯	◯	◯	◯
Non-european caucasian (white)*:	◯	◯	◯	◯
Native American (Autochthon):	◯	◯	◯	◯
African (black):	◯	◯	◯	◯
Asiatic:	◯	◯	◯	◯
Pacific Islander(Autochthon):	◯	◯	◯	◯
Unknown:	◯	◯	◯	◯

Non-european caucasian (white)*: North african origin (Algeria, Tunisia) or the Middle East (Egypt, Iran) or South Asia (Afghanistan, India)

25. What is the **ethnic or cultural** background of **your parents** (who raised you) ?
Check **all that apply**.

	Father	Mother
Canadian:	◯	◯
Native American:	◯	◯
French:	◯	◯
British:	◯	◯
Irish:	◯	◯
Scottish:	◯	◯
American (USA):	◯	◯
Italian:	◯	◯
Greek:	◯	◯
Portuguese:	◯	◯
Haitian:	◯	◯
African:	◯	◯
Latino-American:	◯	◯
Jewish:	◯	◯
Arabic:	◯	◯
Asiatic:	◯	◯
Unknown:	◯	◯
specify: ____ Other :	◯	◯

YOUR MEDICAL HISTORY

Your health status and medical problems interest us. They could help better understand your prostate health.

26. Has a **doctor** ever told you, you had one of the following **diseases** or **pathologies**?

**Prostate diseases:**	Yes	No	Unknown	If yes, **how old** were you when **first diagnosed**
Benign prostatic hyperplasia or **enlarged prostate**	◯	◯	◯	__|__
Acute prostatitis or **prostate infection**	◯	◯	◯	__|__
Chronic prostatitis or **regular prostate pain**	◯	◯	◯	__|__

Urinary bladder or kidney diseases:

	Yes	No	Unknown	If yes, **how old** were you when **first diagnosed**
**Repeated urinary infections** (at least two occurrences).	○	○	○	
Interstitial cystitis or **bladder inflammation**.	○	○	○	
**Renal disease** causing renal insufficiency.	○	○	○	

Check **all that**

If yes,

- ○ Are you under medication?
- ○ Are you under dialysis?
- ○ Are you followed by a specialist (nephrologist)?

Diabetes:

	Yes	No	Unknown	If yes, **how old** were you when **first diagnosed**
Have you been diagnosed with diabetes?	○	○	○	

If yes, can you tell which type of diabetes?

	Yes	Unknown
**Type 1 diabetes**: diagnosed at an early age and always treated with insulin	○	○
**Type 2 diabetes:** diagnosed in adults often overweight or older, sometimes treated with insulin	○	○

If you have diabetes, did you have diabetes related **complications** such as problems with your eyes, your kidneys, your legs or your feet?

Yes ○ No ○ Unknown ○

If yes, could you specify?:

Cardio-vascular disease:

	Yes	No	Unknown	If yes, **how old** were you when **first diagnosed**
**High cholesterol** or blood fats.	○	○	○	|__|__|
Hypertension or **high blood pressure.**	○	○	○	|__|__|
Myocardial infarction or **heart attack.**	○	○	○	|__|__|
Arteriosclerosis or **blood vessels blockage in legs**	○	○	○	|__|__|
Cerebrovascular accident (**CVA**) or stroke	○	○	○	|__|__|
Heart failure or **heart disease** limiting efforts	○	○	○	|__|__|

Gastro-intestinal disease:

	Yes	No	Unknown	If yes, **how old** were you when **first diagnosed**
**Liver disease** such as cirrhosis or Hepatitis A, B, or C	○	○	○	|__|__|
**Stomach disease** such as ulcers	○	○	○	|__|__|
**Inflammatory disease** of the intestine or the **colon** such as ulcerative colitis or Crohn's disease	○	○	○	|__|__|

Other:

	Yes	No	Unknown	If yes, **how old** were you when **first diagnosed**
Inflammation of articulations such as **rheumatoid arthritis** (exclude arthrosis which is joint wearing out)	○	○	○	|__|__|
Bone mass loss such as **osteoporosis**	○	○	○	|__|__|
Chronic respiratory insufficiency such as **emphysema** or **chronic bronchitis**	○	○	○	|__|__|
**Collagen disease** such as epidermolysis, lupus erythematosus, or scleroderma	○	○	○	|__|__|
Hemiplegia or **paralysis** of one or many parts of the body	○	○	○	|__|__|

27. Do you suffer from **diseases other** than those listed?

Yes ○ No ○

If yes, specify

|______________________________|

|______________________________|

28. Have you ever had surgery?

Yes ○ No ○

If yes, which one?

|______________________________|

|______________________________|

29. Have you ever been diagnosed with cancer? Yes ◯ No ◯ Unknown ◯

If yes, please specify: Check all that apply.

- ◯ Lung
- ◯ Colon - Rectum - Intestine
- ◯ Bladder
- ◯ Kidney
- ◯ Testis
- ◯ Leukemia
- ◯ Hodgkin lymphoma
- ◯ Non-Hodgkin lymphoma
- ◯ Stomach
- ◯ Other - specify: ____

If yes, did you have metastasis : Yes ◯ No ◯ Unknown ◯

YOUR FAMILY MEDICAL HISTORY

We are interested in the health of your family members as it could influence yours. In this section, family members are defined as having blood ties with you

30. Have some of **your family** members been diagnosed with **prostate cancer**?

	Yes	No	Unknown	Maternal family	Yes	No	Unknown	Paternal family	Yes	No	Unknown
Father:	◯	◯	◯	Grandfather:	◯	◯	◯	Grandfather:	◯	◯	◯

	Yes	No	Unknown	
Brother(s):	◯	◯	◯	If yes, indicate the **number** of brothers with prostate cancer: ___
Son(s):	◯	◯	◯	If yes, indicate the **number** of sons with prostate cancer: ___
Maternal family Uncle(s):	◯	◯	◯	If yes, indicate the **number** of uncles with prostate cancer: ___
Paternal family Uncle(s):	◯	◯	◯	If yes, indicate the **number** of uncles with prostate cancer: : ___

31. Are there any of the following cancers in your close family, per blood ties ?

If yes, check **under the affected organ** and if applicable, **indicate the number** of concerned persons.

	Yes	No	Unknown	Bladder	Kidney	Testicle	Lung	Pancreas	Colorectal
Father:	○	○	○	○	○	○	○	○	○
Mother :	○	○	○	○	○		○	○	○
Brother(s):	○	○	○	__	__	__	__	__	__
Sister(s):	○	○	○	__	__		__	__	__

Maternal family				Bladder	Kidney	Testicle	Lung	Pancreas	Colorectal
Grandfather:	○	○	○	○	○	○	○	○	○
Grandmother:	○	○	○	○	○		○	○	○

Paternal family				Bladder	Kidney	Testicle	Lung	Pancreas	Colorectal
Grandfather:	○	○	○	○	○	○	○	○	○
Grandmother:	○	○	○	○	○		○	○	○

Continued for the following cancers:

If yes, check **under the affected organ** and if applicable, **indicate the number** of concerned persons.

	Breast	Ovary	Uterine (other than cervix)	Other	Specify
Father:	○			○	________
Mother:	○	○	○	○	________
Brother(s):	__			__	________
Sister(s):	__	__	__	__	________

Maternal family	Breast	Ovary	Uterine (other than cervix)	Other	Specify
Grandfather:	○			○	________
Grandmother:	○	○	○	○	________

Paternal family	Breast	Ovary	Uterine (other than cervix)	Other	Specify
Grandfather:	○			○	________
Grandmother:	○	○	○	○	________

YOUR HISTORY OF TOBACCO USE

SECOND-HAND SMOKE EXPOSURE

We are interested in your exposure to second-hand smoke during childhood and over the last year.

32. During the period **from your birth until age 18**, did you live for more than 1 year with one or many **regular** smokers at home?

Yes ○ No ○

If yes, **for how long** have you been exposed to second-hand smoke at home?

Check **one** answer

- ○ during 1 to 3 years
- ○ during 4 to 6 years
- ○ during 7 to 9 years
- ○ during 10 to 12 years
- ○ during 13 to 15 years
- ○ during 16 to 18 years

There were usually at home:

Check **one** answer

- ○ one smoker
- ○ two smokers
- ○ more than two smokers

---

33. Over **the last year**, for how long on average do you estimate **the number of hours** you have been exposed to second-hand smoke?

At **home** : |__|__| number of hours **/day**

and

elsewhere : |__|__| number of hours**/week**

YOUR HISTORY OF TOBACCO USE

CIGARETTE SMOKING

Smoking cigarette has an impact on your health; we are thus interested in knowing your cigarette smoking habits.

34. Did you smoke **at least 5 packs** of cigarettes (or **100 cigarettes**) over **your whole life**?

Yes ◯ No ◯ → Go to question **41**

If yes how old were you when you started |__|__| years **or** in what year: |__|__|__|__|

35. Do you **presently smoke** cigarettes? Yes ◯ No ◯

If yes, **how many cigarettes** did you smoke per day or per week on average over **the last year**?

|__|__| number of cigarettes**/day** **or** |__|__| number of cigarettes**/week**

36. Have you **stopped** smoking? Yes ◯ No ◯

If yes, how old were you when you stopped? |__|__| years **or** in what year: |__|__|__|__|

Did you stopped smoking and started again? Yes ◯ No ◯

If yes, can you evaluate the total number of years you smoked?

Total number of years: |__|__| years

If yes, can you evaluate how often you have stopped and started smoking again?

Total number of years: |__|__| years

37. Whether you are an active or an ex-smoker, **how many cigarettes** did you smoke on average over **your whole active period**? (**not** only over the last year)

|__|__| number of cigarettes**/day** **or** |__|__| number of cigarettes**/week**

YOUR YOUTH

We are interested in your lifestyle during your teenage years, from age 15 to 18 included.

YOUR ALCOHOL CONSUMPTION

38. From **15 to 18 years old** inclusively, did you drink alcoholic beverages at least once a month? Yes ◯ No ◯

If yes, how **old** were you when **you started to consume** alcoholic beverages? |__|__| years

39. What was **on average** your consumption of the following types of alcoholic beverages during your **teenage years**.

Beer

How many **bottles or cans of beer** (350 ml or 12 ounce cans) did you drink on **average** per week or per month? |__|__| per week **or** |__|__| per month

Spirit

How many **glasses** (1 ounce) **of spirits** did you drink on **average** per week or per month? |__|__| per week **or** |__|__| per month

Wine

How many **glasses of wine** (4 ounces) did you drink on **average** per week or per month? |__|__| per week **or** |__|__| per month

Aperitif

How many **glasses of aperitif** (2 ounces) did you drink on **average** per week or per month? |__|__| per week **or** |__|__| per month

YOUR WEIGHT HISTORY

40. How much did you weigh at age 18 to 20 years old? |__|__|__|.|__| Lb **or** |__|__|__|.|__| Kg

41. Did you ever intentionally lose more than 10 pounds (4.5 kg) that you then regained? Yes ◯ No ◯

If yes, how many times: |__|__|

42. What has been your maximal weight after age 18-20 years? |__|__|__|.|__| Lb **or** |__|__|__|.|__| Kg

YOUR YOUTH

YOUR SPORTS AND PHYSICAL ACTIVITES

43. During your **teenage years** (**age 15 to 18 included**) did you practice a physical activity or a sport?

Yes ◯ No ◯

If yes, could you indicate the **frequency** for the following types of exercises ?

	6 to 7 times per week	3 to 5 times per week	1 to 2 times per week	Less than 1 time per week	Never
**STRENUOUS EXERCISE** **(HEART BEATS RAPIDLY)** i.e.running, jogging, hockey, football, soccer, squash, basketball, cross country skiing, judo, roller skating, vigorous swimming, vigorous long distance bicycling)	◯	◯	◯	◯	◯
**MODERATE EXERCISE** **(NOT EXHAUSTING)** i.e. fast walking, baseball,tennis, easy bicycling, volleyball, badminton, easy swimming, alpine skiing, popular and folk dancing)	◯	◯	◯	◯	◯
**MILD EXERCISE** **(MINIMAL EFFORT)** i.e. yoga, archery, fishing from river band, bowling, horseshoes, golf, snow-mobiling, easy walking)	◯	◯	◯	◯	◯

44. During your **teenage years** (**age 15 to 18 included**), were you an elite or high level athlete (for competition)?

Yes ◯ No ◯

If yes, which sport did you practice? ______________________

How many hours per week on average? |__|__| hours/week

45. During your **teenage years** (**age 15 to 18 included**), were you an amateur sportsman, as a leisure time physical activity?

Yes ◯ No ◯

If yes, which sport did you practice? ______________________

How many hours per week on average? |__|__| hours/week

YOUR ANDROPAUSE

We are interested in evaluating the possibility that you are in andropause.

Over the last year,

	Yes	No	Not applicable
1- Did you feel a **decrease** in your **libido**?	◯	◯	
2- Did you feel a **decrease** in **energy**?	◯	◯	
3- Did you feel a **decrease** in **strength** and/or **stamina**?	◯	◯	
4- Has your **height decreased**?	◯	◯	
5- Did you notice a **decrease** in your "**joie de vivre**"?	◯	◯	
6- Did you feel **sad** or **glum**?	◯	◯	
7- Were your **erections less strong**?	◯	◯	
8- Did you notice it was **more difficult to maintain an erection** until the end of the sexual intercourse?	◯	◯	
9- Did you sleep after meals?	◯	◯	
10- Has your **professional performance decreased**?	◯	◯	◯

Please indicate time at the end : H H : M M
|__|__| : |__|__|

Please check that you answered all questions.

Thank you very much for your participation!

5733038076

1-______

YOUR EXPERIENCE IS A VALUE FOR US!

This questionnaire aims to obtain your comments about your screening tests for prostate cancer. We wish to know your experience in order to improve our practice.

YOUR EXPERIENCE WITH THE BLOOD TEST

The blood test for prostate cancer detection is called Prostate Specific Antigen or PSA. The abbreviation PSA will be used in this questionnaire.

Fill in the circle or make an X for each response: ● ⊗

1. Why did you have a PSA blood test for prostate cancer detection? (check only one answer)
   - ◯ Screening for prostate cancer
   - ◯ Presence of signs or symptoms of a prostate illness
   - ◯ I do not remember

2. Which doctor prescribed you the PSA blood test for prostate cancer detection? (check only one answer)
   - ◯ My family doctor
   - ◯ A family doctor who is not my usual one
   - ◯ My urologist (prostate specialist)
   - ◯ An urologist who is not my usual one
   - ◯ Another specialist doctor
   - ◯ I do not remember

3. Did you discuss with your doctor about the benefits and disadvantages for you of having a PSA blood test for prostate cancer detection? (check only one answer)
   - ◯ Yes, absolutely
   - ◯ Yes, briefly
   - ◯ Not, not at all
   - ◯ I do not remember

4. Think about the decision you took upon meeting that doctor, of having a PSA blood test for prostate cancer detection.

For each of the following statements, check one that corresponds best to your decision

	Totally agree	Somewhat agree	Neither agree nor disagree	Somewhat disagree	Totally disagree
**It was the right decision**	◯	◯	◯	◯	◯
**I regret the choice I made**	◯	◯	◯	◯	◯
**I would make the same choice if I had to**	◯	◯	◯	◯	◯
**That choice was harmful to me**	◯	◯	◯	◯	◯
**It was a wise decision**	◯	◯	◯	◯	◯

YOUR EXPERIENCE WITH THE PROSTATE BIOPSY

5. With which doctor(s) did you decide to have a prostate biopsy? You may check more than one answer if many doctors helped you with that decision

- ◯ My family doctor
- ◯ A family doctor who is not my usual one
- ◯ My urologist (prostate specialist)
- ◯ An urologist who is not my usual one
- ◯ Another specialist doctor
- ◯ I do not remember

6. Think about the decision you took upon meeting that or those doctor(s), to have a prostate biopsy for prostate cancer detection.

For each of the following statements, check one that corresponds best to your decision.

	Totally agree	Somewhat agree	Neither agree nor disagree	Somewhat disagree	Totally disagree
**It was the right decision**	◯	◯	◯	◯	◯
**I regret the choice I made**	◯	◯	◯	◯	◯
**I would make the same choice if I had to**	◯	◯	◯	◯	◯
**That choice was harmful to me**	◯	◯	◯	◯	◯
**It was a wise decision**	◯	◯	◯	◯	◯

7. Did the doctor proceed under local anesthesia to make the biopsy?
(Check only one answer)

- ◯ No
- ◯ Yes
- ◯ I do not remember

8. Please indicate the level of pain you felt when you had the prostate biopsy, on the thermometer, from 0 to 10.

Please blacken the number corresponding to the level of pain.

Example: ■

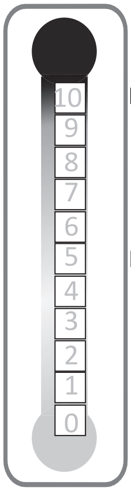

Extreme pain

Moderate pain

No pain

---

9. Please indicate the level of anxiety you felt when you had the prostate biopsy, on the thermometer, from 0 to 10.

Please blacken the number corresponding to level of anxiety.

Example: ■

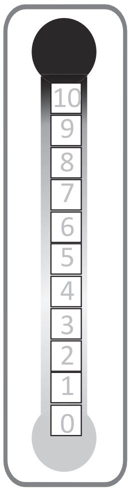

Extreme anxiety

Moderate anxiety

No anxiety

---

10. We listed below side-effects that can occur after a prostate biopsy.
Check if you did or not experience these situations.

	Yes	No
Major rectal bleeding :	◯	◯
Fever necessitating medical advice:	◯	◯
Difficulty with urination:	◯	◯
Urinary infection:	◯	◯
Prostate pain:	◯	◯
Erection problems:	◯	◯
Blood in sperm:	◯	◯

Please indicate time at the end : |__|__| : |__|__|

The whole team thanks you for completing this questionnaire.

QUESTIONNAIRE ON MALE HEALTH

This questionnaire should be completed by the participant himself, without any assistance. The questions concern your sexual intimacy, behavior, and lifestyle.

As you know, our study investigates different lifestyle aspects. These aspects may vary considerably between individuals. We however know that sexual conduct may cause some illnesses. We consider this subject important since it could help prevent prostate cancer. We count on your cooperation.

We would appreciate that you answer all questions, in whole or in part, doing the best you can. We understand that some questions may make you feel uneasy. We wish to remind you that the analysis of the results of this study will be carried out under strict confidentiality.

Thank you for your help.

Please indicate time at the beginning : H H : M M ___ ___ : ___ ___

Section à remplir par le superviseur: Questionnaire complété le : 2 0 1 _ - _ _ - _ _ Sous la supervision de: Initiales _ _ _ _

OBLIGATOIRE

Initiales pour l'informatisation

3- _ _ _ _ _

1475243073

YOUR SEXUALITY

The following section of the questionnaire concerns your sexuality, please answer as sincerely as possible. We understand that some questions may make you feel uneasy. We wish to remind you that the analysis of the results of this study will be carried out under strict confidentiality.

Please do your best to answer all questions. An estimate will be more useful for our study than a blank response.

Fill in the circle or make an X for each response: ● ⊗

SECTION ON YOUR PRESENT SITUATION

A couple is herein defined as 2 persons of the same sex or of opposite sex, having or not sexual relationships.

1.- Do you **currently** live as a couple? Yes ○ No ○

If yes, for **how many years** have you lived as a couple **with the same person**?

Number of years: |__|__| years

If no, have you ever lived as a couple ? Yes ○ No ○

2.- What was the **longest** duration of your former couple relationschip?

Number of years (or months, if less than a year) ○ Check here if not applicable

Number of years: |__|__| years Number of months: |__|__| months

3.- Are you **currently** sexually active? Yes ○ No ○

If yes and if you live in a couple:

○ Do you have sexual intercourse only **within your couple?**

○ Do you have sexual intercourse only **outside your couple?**

○ Do you have sexual intercourse **both within your couple and outside your couple?**

YOUR HISTORY OF SEXUAL CONDUCT

4.- Have you been **circumcised**? Yes ◯ No ◯ → **If no**, go to question **5**

a) If yes, at what age were you circumcised? ◯ At birth **or** |__|__| years ◯ Do not know

b) Why have you been circumcised?

- ◯ At birth, because it was then routinely done at the time
- ◯ For religious reasons
- ◯ Because of repeated infections
- ◯ For aesthetic reasons
- ◯ Other-Specify: |________________|

c) Were you circumcised **before** your **first sexual intercourse?** Yes ◯ No ◯ Do not know ◯

Or

d) Were you circumcised **after** your **first sexual intercourse?** Yes ◯ No ◯ Do not know ◯

5.- Have you been **vasectomized**? Yes ◯ No ◯ → **If no**, go to question **6**

a) If yes, in what year? |__|__|__|__|

Or at what age? |__|__| years

b) Did you have a **second vasectomy**? Yes ◯ No ◯ → **If no**, go to question **6**

c) **If yes**, in what year did you have a second vasectomy? |__|__|__|__|

Or at what age? |__|__| years

6.- Could you indicate your use of **condoms** on a timescale?
Check only **one answer** per **age category:**

a) **Did you have sexual intercourse** during the following periods of your life?

Periods of your life					
15 - 19	20 - 29	30 - 39	40 - 49	50 - 59	More than 60
Yes ◯	Yes ◯	Yes ◯	Yes ◯	Yes ◯	Yes ◯
No ◯	No ◯	No ◯	No ◯	No ◯	No ◯
Do not know ◯	Do not know ◯	Do not know ◯	Do not know ◯	Do not know ◯	Do not know ◯

b) **If you had sexual intercourse:**

1) **Did you use condoms** during the following periods of your life?

Periods of your life					
15 - 19	20 - 29	30 - 39	40 - 49	50 - 59	More than 60
Never ◯	Never ◯	Never ◯	Never ◯	Never ◯	Never ◯
Sometimes ◯	Sometimes ◯	Sometimes ◯	Sometimes ◯	Sometimes ◯	Sometimes ◯
Often ◯	Often ◯	Often ◯	Often ◯	Often ◯	Often ◯
Do not know ◯	Do not know ◯	Do not know ◯	Do not know ◯	Do not know ◯	Do not know ◯

2) Did you use condoms **with your stable partner** during the following periods of your life?

Periods of your life					
15 - 19	20 - 29	30 - 39	40 - 49	50 - 59	More than 60
Yes ◯	Yes ◯	Yes ◯	Yes ◯	Yes ◯	Yes ◯
No ◯	No ◯	No ◯	No ◯	No ◯	No ◯
Do not know ◯	Do not know ◯	Do not know ◯	Do not know ◯	Do not know ◯	Do not know ◯
Not applicable ◯	Not applicable ◯	Not applicable ◯	Not applicable ◯	Not applicable ◯	Not applicable ◯

3) Did you use condoms **with occasional partners** during the following periods of your life?

Periods of your life					
15 - 19	20 - 29	30 - 39	40 - 49	50 - 59	More than 60
Yes ◯	Yes ◯	Yes ◯	Yes ◯	Yes ◯	Yes ◯
No ◯	No ◯	No ◯	No ◯	No ◯	No ◯
Do not know ◯	Do not know ◯	Do not know ◯	Do not know ◯	Do not know ◯	Do not know ◯
Not applicable ◯	Not applicable ◯	Not applicable ◯	Not applicable ◯	Not applicable ◯	Not applicable ◯

7.- When you were using a condom, it was generally :

With your **stable partner** for

◯ All the duration of the sexual act
◯ A part of the sexual act
◯ Not applicable

With your **occasional partners** for

◯ All the duration of the sexual act
◯ For a part of the sexual act
◯ Not applicable

8.- Please indicate the total **number of ejaculations per month** resulting of masturbation or complete sexual intercourse in the following table :

For each period of your life, indicate the number of times each month.
Indicate **0** if none and write an **X** if you do not remember.

	Periods of your life					
	15 - 19	20 - 29	30 - 39	40 - 49	50 - 59	more than 60
By masturbation per month:	|__|__|	|__|__|	|__|__|	|__|__|	|__|__|	|__|__|
By sexual intercourse per month:	|__|__|	|__|__|	|__|__|	|__|__|	|__|__|	|__|__|

YOUR HISTORY OF SEXUAL RELATIONSHIPS

RELATIONSHIP WITH WOMEN

9.- Have you ever had sexual intercourse with a woman? Yes ◯ No ◯ → **If no** go to question **19**

10.-**How old** were you when you had sexual intercourse with a woman for the first time? |__|__| years

11.- How many female sexual partners (including prostitutes) have you had in your life? (regular and casual)

- ◯ 1
- ◯ 2-5
- ◯ 6-10
- ◯ 11-20
- ◯ 21-50
- ◯ 51-100
- ◯ More than 100
- ◯ Check here if difficult to answer

12.- Have you ever had **anal sex** with a woman? [Your penis in the anus (rectum) of a woman]

Yes ◯ No ◯ ⇒ **If no** go to question **14**

13.- How often?

Occasionally	Often	Most of the time
◯	◯	◯

14.- How **old** were you when you had anal sex with a woman for the first time? |__|__| years

---

15.- Have you ever had sex with a female prostitute?

Yes ◯ No ◯ ⇒ **If no** go to question **19**

16.- **How many** female prostitutes have you had sexual contact with?

- ◯ 1
- ◯ 2-5
- ◯ 6-10
- ◯ 11-20
- ◯ 21-50
- ◯ 51-100
- ◯ More than 100
- ◯ Check here if difficult to answer

17.- Have you ever had **anal sex** with a **female prostitute**? [Your penis in the anus (rectum) of the woman]

Yes ◯ No ◯ ⇒ **If no** go to question **20**

18. How often?

Occasionally	Often	Most of the time
◯	◯	◯

19.- **How old** were you when you had anal sex with a female prostitute for the first time? |_|_| years

RELATIONSHIP WITH MEN

20.- Have you ever had sexual intercourse with a man? Yes ◯ No ◯ ⇒ **If no** go to question **31**

21.- How old were you when you had sexual intercourse with a man for the first time? |_|_| years

22.- How many male sexual partners (including prostitutes or transvestites) have you had in your life? (regular and casual)

- ◯ 1
- ◯ 2-5
- ◯ 6-10
- ◯ 11-20
- ◯ 21-50
- ◯ 51-100
- ◯ More than 100
- ◯ Check here if difficult to answer

23.- Have you ever had anal sex with a man? [Your penis in the anus (rectum) of a man]

Yes ◯ No ◯ ⇒ **If no** go to question **26**

24. How often?

Occasionally	Often	Most of the time
◯	◯	◯

25.- **How old** were you when you had anal sex with a man for the first time? |__|__| years

26.- Have you ever had sex with a male homosexual or bisexual prostitute or a male transvestite?

Yes ◯ No ◯ ⟹ **If no** go to question **30**

27.- **How many** male homosexual or bisexual prostitutes or male transvestites have you had sexual contact with?

- ◯ 1
- ◯ 2-5
- ◯ 6-10
- ◯ 11-20
- ◯ 21-50
- ◯ 51-100
- ◯ More than 100
- ◯ Check here if difficult to answer

28.- Have you ever had anal sex with a homosexual or bisexual prostitute or a male transvestite? [Your penis in the anus (rectum) of a man]

Yes ◯ No ◯ ⟹ **If no** go to question **31**

29.- How old were you when you had anal sex with a male homosexual or bisexual prostitute or a male transvestite for the first time?

|__|__| years

30. How often?

Occasionally	Often	Most of the time
◯	◯	◯

INTERCOURSE WITH A WOMAN OR A MAN

31.- When having sexual intercourse with a woman or a man, have you ever been penetrated into the anus (rectum)?

Yes ◯ No ◯ ⟹ **If no** go to question **32**

32. How often?

Occasionally	Often	Most of the time
◯	◯	◯

SECTION ON GENITAL OR SEXUALLY TRANSMITTED DISEASES

33.- Have you ever had a sexually transmitted infection or disease?

Yes	No	Do not know
○	○	○

If yes, has a doctor ever told you that you had one of the following infections or diseases:

	Yes	No	Do not know
Infection with genital Trichomonas	○	○	○
Genital warts, condylomas, human papilloma viruses	○	○	○
Chlamydia	○	○	○
Genital herpes	○	○	○
Syphilis	○	○	○
Gonorrhea	○	○	○
Genital ulcers	○	○	○
AIDS or HIV	○	○	○
Hepatitis B	○	○	○
Ureaplasma hominis	○	○	○
Others	○	○	○

34.- As far as you remember, has any of your **sexual partners** had sexually transmitted diseases :

Yes	No	Do not know
◯	◯	◯

If yes, do you remember which disease(s) he or she had?

	Yes	No	Do not know
Infection with genital Trichomonas	◯	◯	◯
Genital warts, condylomas, human papilloma viruses	◯	◯	◯
Chlamydia	◯	◯	◯
Genital herpes	◯	◯	◯
Syphilis	◯	◯	◯
Gonorrhea	◯	◯	◯
Genital ulcers	◯	◯	◯
AIDS or HIV	◯	◯	◯
Hepatitis B	◯	◯	◯
Ureaplasma hominis	◯	◯	◯
Others	◯	◯	◯

Please indicate time at the end : H H : M M |_|_| : |_|_|

This is the end of the questionnaire.
Please, take a moment to revise your answers in all sections of the questionnaire.

Thank you very much for your participation!

DIETARY SUPPLEMENTS OR MEAL REPLACEMENTS

1. Do you take, on a regular basis, meal replacement or nutritional supplement such as Boost, Ensure, Glucerna, Nutribar, or others?

Yes ◯ No ◯

If yes, what is (are) the brand name(s) of the supplements you take?

◯ Boost
◯ Ensure
◯ Glucerna
◯ Other, specify ________

If yes, what type of product(s) do you take?

◯ Regular
◯ Hyperproteinated
◯ High-calorie (about 360 Cal)
◯ Low-calorie (about 200 Cal)
◯ Low-sugar for diabetics
◯ Other, specify ________

2. What quantity of solid or liquid product do you take every time you use meal replacement or nutritional supplement?

LIQUID SUPPLEMENT

◯ Less than ½ bottle
◯ ½ bottle
◯ 1 bottle
◯ 2 bottles
◯ More than 2 bottles

BAR

◯ Less than ½ bar
◯ ½ bar
◯ 1 bar
◯ 2 bars
◯ More than 2 bars

Other, specify ________

3. How often have you taken a meal replacement or nutritional supplement over the 4 last weeks?

◯ Everyday
◯ 5-6 times a week
◯ 3-4 times a week
◯ Twice a week
◯ Once a week
◯ Less than once a week

Initiales pour l'informatisation

8136345190
