# Supplementary material for: Cohort profile: BioCaPPE (Biomarkers of Prostate Cancer/Prevention and Environment) – a Canadian multicentre prospective study of lifestyle and candidate biomarkers in relation to prostate cancer risk
Source: BMJ Open. 2026 May 28;16(5):e111118. doi: 10.1136/bmjopen-2025-111118 (PMC13223634; doi:10.1136/bmjopen-2025-111118)
Supplement: Supplementary file 2 [file bmjopen-2025-111118.reviewer_comments.pdf]

PEER REVIEW HISTORY

BMJ Open publishes all reviews undertaken for accepted manuscripts. Reviewers are asked to complete a checklist review form and are provided with free text boxes to elaborate on their assessment. These free text comments are reproduced below.

ARTICLE DETAILS

Title (Provisional)

Cohort Profile: BioCaPPE (Biomarkers of Prostate Cancer/Prevention and Environment)—A Canadian Multicenter Prospective Study of Lifestyle and Candidate Biomarkers in Relation to Prostate Cancer Risk.

Authors

Moussa, Hanane; Tourigny, Roxane; Robitaille, Karine; Bussières, Vanessa; Fadel, Jonathan; Ben Souilah, Farah; Diabaté, Lamoussa; Hovington, Hélène; Bettan, Laurence; Lacombe, Louis; Dujardin, Thierry; Lodde, Michele; Toren, Paul; Aprikian, Armen; Saad, Fred; Carmel, Michel; Jeldres, Claudio; Lamarche, Benoit; Bergeron, Alain; BIOCaPPE, Network; Fradet, Yves; Fradet, Vincent

---

VERSION 1 - REVIEW

**Reviewer**	**1**
**Name**	**Holmberg, Lars**
**Affiliation**	**Prof. of Cancer Epidemiology**
**Date**	**04-Dec-2025**
**COI**	**None**

A cohort profile from this study invites constructive collaborations and provides important background information about the study for readers of future publications.

The text could be made more accessible and the information more complete by considering the following:

1. The presentation is somewhat unfocused and in places deals with subjects not relevant for understanding the core elements of the two cohorts. Examples are textbook statements about prostate cancer (e.g. the two first paragraphs of the introduction). There are also discussions about findings in other studies/cohorts under "Relevance…" that rather belong to future publications about specific findings.

2. There is a lack of consistency between the aims as presented line 110-116 and later information and comments about future perspectives. E.g. on line 380 it is stated that the study offers an alternative to prostate cancer screening rather than being a prostate cancer prevention study. A screening study is quite another matter. On line 424 quality of life and

decision-making is introduced as a goal of the study, also quite another matter and requiring another study design. These are examples that the text could be more focused on factual information about the cohorts rather than branching out to too far-reaching speculations about which information the study can provide.

3. While the text could be more informative by being substantially shorter in some parts, there are some central aspects that now are missing. The first is a thorough motivation for the study design considering our current understanding of prostate cancer natural history. By choosing these two cohorts, most (if not all) men with incident prostate cancer within the first three to five years after inclusion must have had the cancer established already at enrollment. It is then a challenge to motivate that recent e.g. physical activity and dietary habits is informative for prostate cancer prevention.

4. Another central aspect is statistical precision. Most clues we have for the relation between modifiable lifestyle habits and prostate cancer risk indicate weak relationships. Then to investigate the link the impact of lifestyle factors on biomarkers and then to prostate cancer will be statistically very challenging unless studies are very large. This study is in this context moderately large and the number of cases to date only 314. The cohort profile needs concrete evaluation of the statistical precision for some typical, realistic hypotheses.

5. The items under 3 and 4 above probably also need be mentioned under strengths and limitations.

6. The abstract will have to be changed if/when the comments above are tackled

Please note: the BMJ checklist is not adopted specifically to cohort profiles and I have used it as an indication only to where revisions could be considered.

**Reviewer**	**2**
**Name**	**Venclovas, Zilvinas**
**Affiliation**	**Lithuanian University of Health Sciences**
**Date**	**17-Dec-2025**
**COI**	**None**

How many patients from each group underwent prostate MRI testing, and what were the results?

Given the statistically significant differences in PSA levels, the rate of prostate biopsy history (PBH), and age, how do you account for these discrepancies between the two groups?

Considering the slower development of prostate cancer, why was a two-year follow-up period chosen, and do you believe a longer follow-up would yield different insights?

What is the PSA density for both groups, and could the differences in PBH contribute to the variations in PSA levels observed?

With a notable difference in the number of cardiovascular events between the groups, could the comorbidities in Group 2 be a significant factor influencing the decision against performing a prostate biopsy?

What explanations can you provide regarding the differences in financial status between the two groups of patients? How might this impact their healthcare decisions?

What types of biopsies were performed on the patients—were they systemic, MRI-guided, or both?

Why did Group 2 have a higher incidence of ISUP2? Were the biopsies conducted after MRI assessments, and could that influence the ISUP grading?

What factors influenced the decision to conduct a second biopsy for patients in the first group, and what criteria were used to determine the timing?

These questions can guide a deeper discussion and clarification from the authors regarding the methodology and findings of their study.

VERSION 1 - AUTHOR RESPONSE

Reviewer #1 Comments

1. The presentation is somewhat unfocused and in places deals with subjects not relevant for understanding the core elements of the two cohorts. Examples are textbook statements about prostate cancer (e.g. the two first paragraphs of the introduction). There are also discussions about findings in other studies/cohorts under "Relevance…" that rather belong to future publications about specific findings.

As suggested, a substantial part of the two first paragraphs of the introduction has been removed, and the text has been updated at lines 77 to focus on topics relevant for understanding the cohorts. In the Relevance section, the *Comparison with Other Similar Studies* section (last paragraphs) has been fully removed, which was less relevant for understanding the core elements of the cohort.

2. There is a lack of consistency between the aims as presented line 110-116 and later information and comments about future perspectives. E.g. on line 380 it is stated that the study offers an alternative to prostate cancer screening rather than being a prostate cancer prevention study. A screening study is quite another matter. On line 424 quality of life and decision-making is introduced as a goal of the study, also quite another matter and requiring another study design. These are examples that the text could be more focused on factual information about the cohorts rather than branching out to too far-reaching speculations about which information the study can provide.

We agree that screening and decision-making are outside the scope of this study and have focused the text accordingly. The primary objective of this study is to evaluate the association between lifestyle-related candidate biomarkers and the risk of prostate cancer (updated

manuscript lines 93-94). Yet our study has the potential to lay the conceptual foundations for possible future screening studies by introducing new and innovative biomarkers.

We modified the text at lines 463-468 to better reflect this:

"The biomarkers evaluated in this study are standardized low-cost tests performed in most laboratories. However, to our knowledge, it is the first time they are formally evaluated in relation with PCa risk. Through the identification of novel biomarkers, we aim to lay the conceptual foundations to develop a new methodology for assessing PCa risk. These modifiable biomarkers could also represent a significant conceptual advancement potentially applicable to cancer prevention strategies for the general population."

Quality of life assessment was introduced in this study as an *a priori* secondary objective of the BIOCaPPE study (lines 98-99). The methodology was well described (lines 233-244), including the questionnaire used to measure patient experience (lines 252-254).

We improved clarity for this methodology (lines 252-254):

"Experience <u>and decisional regret</u> related to PSA testing and prostate biopsy were captured using a questionnaire adapted from PiCTure Study[36] (see Supplementary Material)."

We have also modified the Relevance and Impact section manuscript to clarify the observational nature quality-of-life assessments (lines 482-490):

"From a practical standpoint, assessing quality of life in relation to modifiable lifestyle factors and PCa incidence introduces a human-centered dimension. The relevance of this dimension has been previously highlighted[64 65] and is considered essential in decision-making processes during prostate cancer risk assessment and during active surveillance. Clinical decisions are now recommended to use a shared approach between physician and patient, with a quality-of-life considerations playing a central role[11-14]. Thus, using validated questionnaires, we herein propose a comprehensive observational evaluation that will evolve over an extended period of follow-up and provide an opportunity to explore human-centered factors in the context of prostate cancer risk assessment."

3. While the text could be more informative by being substantially shorter in some parts, there are some central aspects that now are missing. The first is a thorough motivation for the study design considering our current understanding of prostate cancer natural history. By choosing these two cohorts, most (if not all) men with incident prostate cancer within the first three to five years after inclusion must have had the cancer established already at enrollment. It is then a challenge to motivate that recent e.g. physical activity and dietary habits is informative for prostate cancer prevention.

We agree that focussing on the main study topics (Comments #1 and #2) is relevant and provides more space for other important topics, such as the one underlined here.

We have added this text to the Relevance, Study Impact and Future Directions section (lines 413-428):

"The context of this study makes it particularly actionable as it recruited participants referred to or at charge of urologists because identified at elevated risk of prostate cancer. Given the typically longer latency of most prostate cancer cases, it is possible that the lifestyle factors (and related biomarkers) may have influenced prostate cancer before this study measured

them. This is in part why we expanded the follow-up duration without restriction. In the same line, this study also measures some factors thought to affect metabolism during teenage years, a period hypothesized to affect prostate growth and possibly dysregulation.
Nevertheless, prostate cancer is known as a complex multifactorial disease[44]. Thus, expanding on Bradford Hill criteria evaluating causality[45], and based on the multicausality framework[46 47], this study aims to identify risk factors (e.g. biomarkers) associated to component causes (e.g. lifestyle element). Even if these are identified somewhat "downstream" or closer to disease identification, they still can be considered a contributing factor to causing the disease of interest. What is more, the overarching hypothesis of this study is that the referral and recruitment context in urology clinics makes the study findings actionable for prostate cancer prevention such as trials testing targeted risk-reduction strategies and patient education about their lifestyle."

4. Another central aspect is statistical precision. Most clues we have for the relation between modifiable lifestyle habits and prostate cancer risk indicate weak relationships. Then to investigate the link the impact of lifestyle factors on biomarkers and then to prostate cancer will be statistically very challenging unless studies are very large. This study is in this context moderately large and the number of cases to date only 314. The cohort profile needs concrete evaluation of the statistical precision for some typical, realistic hypotheses.

We added this to the Strenghts and limitations section (lines 543-555)

"Statistical precision may be limiting the capacity of this study to detect associations of small magnitude. We quantified the statistical power for representative hypotheses involving biomarkers assessed in our cohort. Given the lack of published data defining a clinically meaningful incidence rate ratio for prostate cancer associated with the candidate circulating biomarkers, we based our calculations on statistically meaningful hazard ratios (HRs) used in previous studies. Using the COXREG procedure in SAS (alpha = 5%), the observed number of prostate cancer events (n = 314), and a conservative R^2 value of 0.20 (to account for correlation between adjustment variables), we evaluated HRs ranging from 0.1 to 1.5. Under these assumptions, the minimum detectable HR is 0.82, corresponding to a statistical power of 88.2% (see Table R1 below).

These calculations demonstrate that the cohort achieves sufficient statistical precision to detect realistic, biologically plausible associations for the biomarkers examined. Similar calculations will be repeated for each new analysis to specify the actual power to the updated data."

For the reviewer, here is an example with current data (which will change over time):

Table R1. Hasard ratio (HR) and statistical power

HR	Statistical power
0,7	100,0
0,8	94.3
**0,82**	**88.2**
0,9	38.6
1,1	32.7

1.2	84.4
**1,21**	**85.6**
1.3	98.6
1.4	100
1.5	100

5. The items under 3 and 4 above probably also need be mentioned under strengths and limitations.

We made changes to the Strengths and Limitations section and other sections, when more aligned with text flow.

6. The abstract will have to be changed if/when the comments above are tackled.

We addressed all of the reviewer's comments throughout the revised manuscript. However, we do not believe that changes to the abstract are required. The revisions did not alter the study objectives, methods, or main findings in a way that would impact the accuracy or integrity of the abstract. Therefore, we kept the abstract unchanged.

Reviewer #2 Comments

1-How many patients from each group underwent prostate MRI testing, and what were the results?

As mentioned in the original manuscript, prostate MRI testing was not part of the Canadian standard diagnostic pathway during study design, and the majority of recruitment period. Thus, MRI was left at discretion of treating urologist. MRI data was collected when available.

We added clarifications to reflect these precisions in the Clinical follow-up section at lines 183-189.

"Prostate imaging with ultrasound only without biopsy was allowed but was not performed by clinicians in this study. Prostate imaging with magnetic resonance imaging (MRI), which progressively became available during the recruitment of this study, was ordered as a shared decision with the patient at the discretion of the treating urologist based on clinical preferences and absence of MRI contraindications such as non-MRI-compatible implanted devices, ferromagnetic foreign bodies or claustrophobia. MRI data was collected when available."

We also added this to the Findings to Date section (lines 383-384):

"MRI-guided biopsies were used in 23% of PCa diagnoses, with no difference between Groups 1 and 2 nor in ISUP distribution."

2-Given the statistically significant differences in PSA levels, the rate of prostate biopsy history (PBH), and age, how do you account for these discrepancies between the two groups?

Indeed, Group 1 participants had a recent first negative biopsy, while group 2 participants did not have a previous prostate biopsy. A previous prostate biopsy (except within 6 months of enrollment for Group 1) was an exclusion criterion.

We now emphasize this in the Cohort Description section (line 107).

We consider that this aspect is a strength of the BioCaPPE study, applying the same methodology across two prostate cancer risk scenarios that are common in urology clinics. This allows to explore if the exposure has a similar effect across groups.

We added the following text to the Statistical Analysis section (lines 278-284):

"As expected, the incidence of prostate cancer diagnosis is higher in group 2. Thus, analyses of the whole cohort will be stratified by group. If a similar risk ratio is observed for the exposure variable (e.g. baseline biomarker) with the prostate cancer incidence outcome, then both groups will be combined. If not, stratified analyses will be presented. Also, except for cases when the variable is a mediator in the expected causal pathway based on directed acyclic graphs, all analyses will adjust for age and baseline PSA level as these are established prostate cancer risk factors."

3-Considering the slower development of prostate cancer, why was a two-year follow-up period chosen, and do you believe a longer follow-up would yield different insights?

We clarified this rationale and follow-up to improve clarity and precision (lines 172-182):

"The initial protocol for Group 1 included a recommendation for a prostate biopsy two years after enrollment, based on the Reduction by Dutasteride of Prostate Cancer Events (REDUCE) trial[10], a large-scale trial available at that the time of BioCaPPE study design. However, shortly after the study's launch, the practice of routine re-biopsy at two years for men with a previously negative result was progressively discontinued by clinicians. Instead, an individualized assessment was conducted for all participants based clinical guidelines[11-14] and considering lower urinary tract symptoms, PSA kinetics, digital rectal examination of the prostate, familial cancer history, age, estimated remaining lifespan. The decision to order a prostate biopsy was a shared decision with the participant at the discretion of the treating urologist based on an elevated suspicion of prostate cancer upon follow-up."

Also, we clarified the text to better outline the study design relevance (lines 407-412):

"To our knowledge, BioCaPPE represents the largest multicentric prospective cohort of Canadian men at risk of PCa. This study was initiated slightly before practice changes and eventually clinical guidelines recommending a more selective usage of prostate biopsy. This modern approach involves the patient if a shared decision-making, and considers numerous clinical factors listed above such as symptoms, PSA kinetics, physical examination and familial cancer history."

We added text to the Relevance section to clarify the author's intentions in designing this study (lines 413-428):

"The context of this study makes it particularly actionable as it recruited participants referred to or at charge of urologists because identified at elevated risk of prostate cancer. Given the typically longer latency of most prostate cancer cases, it is possible that the lifestyle factors (and related biomarkers) may have influenced prostate cancer before this study measured them. This is in part why we expanded the follow-up duration without restriction. In the same line, this study also measures some factors thought to affect metabolism during teenage years, a period hypothesized to affect prostate growth and possibly dysregulation.

Nevertheless, prostate cancer is known as a complex multifactorial disease[44]. Thus, expanding on Bradford Hill criteria evaluating causality[45], and based on the multicausality framework[46] [47], this study aims to identify risk factors (e.g. biomarkers) associated to component causes (e.g. lifestyle element). Even if these are identified somewhat "downstream" or closer to disease identification, they still can be considered a contributing factor to causing the disease of interest. What is more, the overarching hypothesis of this study is that the referral and recruitment context in urology clinics makes the study findings actionable for prostate cancer prevention such as trials testing targeted risk-reduction strategies and patient education about their lifestyle."

4-What is the PSA density for both groups, and could the differences in PBH contribute to the variations in PSA levels observed?

The baseline PSA density is only available for Group 1 as only these participants had an ultrasound-guided prostate biopsy session to estimate prostate volume, and no other prostate imaging was mandated at baseline in this study. PSA density information is not available for Group 2 participants who were excluded if they had a previous prostate biopsy. Thus, a between-group comparison of PSA density is not possible in this study.

Please also see our response and text clarifications to the second comment from this reviewer. As expected, previous prostate biopsy history certainly affects the risk of incident prostate cancer in this study (as in other studies) as shown in Figure 2B.

We added this clarification to the data collection procedures (line 159-161):

"Baseline PSA was defined as the last PSA test result before enrollment for all patients with the specification that it must be before prostate biopsy session for Group 1 participants."

5-With a notable difference in the number of cardiovascular events between the groups, could the comorbidities in Group 2 be a significant factor influencing the decision against performing a prostate biopsy?

The history of cardiovascular events is indeed statistically different across groups, and it is possible that they may influence the decision about performing a prostate biopsy, although these conditions are relatively rare (5-7% or lower) making them unlikely to be a significant factor in our models. Nevertheless, we performed additional modeling to test that hypothesis.

We added text to the Results section (lines 319-327):

"Given the significant difference between groups at baseline in some cardiovascular comorbidities, we tested the hypothesis that these comorbidities may be used by clinicians to decide against performing a prostate biopsy. None of the baseline comorbidities were individually associated with time to a prostate biopsy session (all p>0.5). We also tested a common definition of major cardiovascular events (MACE) in cardiovascular trials, where either myocardial infarction or stroke is considered exposed. MACE was also not associated with risk of prostate biopsy (HR 1.12, 95%CI 0.86-1.47, p=0.42). It is thus unlikely that cardiovascular comorbidities was a significant factor influencing the biopsy decision."

As in most jurisdictions, in the province of Québec, prostate biopsy procedures are performed under local anesthesia, rather than general anesthesia. Therefore, cardiac clearance to perform a prostate biopsy is only occasionally required to stop anticoagulant or antiplatelet therapies in complex cases.

6-What explanations can you provide regarding the differences in financial status between the two groups of patients? How might this impact their healthcare decisions?

We added this text to the Findings to date section (lines 333-345):

"Income differed across study groups (chi-squared p = 0.03). Amongst patients recruited at CHUQc-UL (Quebec City), 62% were in Group 1 and 38% in Group 2. At CHUS (Sherbrooke), 94% were in Group 1 and 6% in Group 2. When both Montreal sites are aggregated (CHUM and CUSM), 81% were in Group 1 and 19% in Group 2. Regional differences in annual income are observed in the general population. Similar to the Chi-square test presented in Table 1, in a crude multinomial model, the overall effect of group on annual income was statistically significant (p = 0.035). After adjusting for city, the overall effect of group was no longer significant (p = 0.293), and the odds ratios for Group 2 vs Group 1 shifted toward the null. Specifically, the odds ratio for income category 2 ($60,000-$100,000) vs 1 (<$60,000) decreased from 1.37 to 1.22 (-11.2%), and the odds ratio for income category 3 (>$100,000) vs 1 decreased from 1.14 to 1.05 (-7.5%). These changes indicate that city acts as a confounder of the association between group and income, particularly for the comparison between income categories 2 and 1."

We also added this text to the Strengths and Limitations section (lines 528-534):

"The observed between-group income differences are probably attributable to variations in recruitment proportions among the different cities. They are unlikely to impact healthcare decisions because Canada has a universal health care system. However, annual income can impact lifestyle-related decisions, such as eating higher-quality food elements and practicing physical activity. As a result, we will adjust all models testing lifestyle-related questions by Center/city and income level. We will also test for interaction, when relevant."

7-What types of biopsies were performed on the patients—were they systemic, MRI-guided, or both?

For Group 1 enrollment, all baseline prostate biopsy sessions were performed as systematic ultrasound-guided procedures. In our study, 0.2% of enrollment biopsies were limited to only two zones, not considered systematic. None were MRI-guided. PCa diagnosis was also mainly

determined through transrectal ultrasound-guided prostate biopsy. MRI was slowly integrated into clinical practice during the study and was used in 23% of PCa diagnoses with no difference between Groups 1 and 2 nor in ISUP distribution.

To clarify this aspect, we have added the following to the manuscript:

- PCa incidence and Imaging section (line 202): "PCa incidence was determined mainly through systematic transrectal ultrasound-guided prostate biopsy, which was the guideline-recommended method for pathological diagnosis and grading of PCa at the time of recruitment[12 15 16].
- Lines 383-384: MRI-guided biopsies were used in 23% of PCa diagnoses, with no difference between Groups 1 and 2 nor in ISUP distribution.

8-Why did Group 2 have a higher incidence of ISUP2? Were the biopsies conducted after MRI assessments, and could that influence the ISUP grading?

As mentioned above in response to the second comment from this reviewer, MRI-guided biopsies were only used in 23% of PCa diagnoses, with no difference between Groups 1 and 2 nor in ISUP distribution. Since participants in Group 1 had a first negative biopsy at the time of enrollment, the already larger tumors were identified before enrollment. This was not the case for participants in Group 2. Prostate cancers diagnosed in Group 1 had likely developed or grown more recently than in Group 2. This is highlighted by the fact that half of the cases diagnosed in Group 1 were ISUP Grade group 1 compared to only 31% in Group 2.

9-What factors influenced the decision to conduct a second biopsy for patients in the first group, and what criteria were used to determine the timing?

We clarified the Clinical Follow-up section (lines 172-182):

"The initial protocol for Group 1 included a recommendation for a prostate biopsy two years after enrollment, based on the Reduction by Dutasteride of Prostate Cancer Events (REDUCE) trial[10], a large-scale trial available at that the time of BioCaPPE study design. However, shortly after the study's launch, the practice of routine re-biopsy at two years for men with a previously negative result was progressively discontinued by clinicians. Instead, an individualized assessment was conducted for all participants based clinical guidelines[11-14] and considering lower urinary tract symptoms, PSA kinetics, digital rectal examination of the prostate, familial cancer history, age, estimated remaining lifespan. The decision to order a prostate biopsy was a shared decision with the participant at the discretion of the treating urologist based on an elevated suspicion of prostate cancer upon follow-up."

10-These questions can guide a deeper discussion and clarification from the authors regarding the methodology and findings of their study.

We agree and proceeded accordingly (above).

VERSION 2 - REVIEW

Reviewer 1

**Name**	**Holmberg, Lars**
**Affiliation**	**Prof. of Cancer Epidemiology**
**Date**	**20-Mar-2026**
**COI**	

The authors have made the presentation much clearer and concentrated on the factual presentation of the cohort and the data collection. Thus, the ms is now much more informative as background for analytical studies and for inviting collaboration.
